# Quantifying Chemically Modified Acetylation Induced Changes in the Plant Secondary Cell Wall Structure and Dynamics

**DOI:** 10.1021/acs.biomac.5c00391

**Published:** 2025-08-21

**Authors:** Murtaza Barkarar, Daipayan Sarkar, Christopher G. Hunt, Josh V. Vermaas

**Affiliations:** † Department of Biochemistry and Molecular Biology, 3078 Michigan State University, 612 Wilson Road, East Lansing, Michigan 48824, United States; ‡ MSU-DOE Plant Research Laboratory, 3078 Michigan State University, 612 Wilson Road, East Lansing, Michigan 48824, United States; § Forest Products Laboratory, USDA Forest Service, Madison, Wisconsin 53726, United States; ∥ Department of Biochemistry and Molecular Biology, 3078 Michigan State University, 612 Wilson Road, East Lansing, Michigan 48824, United States

## Abstract

Wood that is resistant to fungal degradation is highly desirable, and chemical acetylation is one way to inhibit wood decay. The open question is whether acetylation inhibits decay strictly by reducing moisture content within the cell wall or if specific interactions with the acetyl group hinder motion within the cell wall and further inhibit decay. We investigate these hypotheses through molecular simulation, acetylating exposed hemicellulose and lignin hydroxyl groups in a secondary plant cell wall model. By comparing diffusion within the model, we find that acetyl group interactions alone do not account for reduced transport, with only modest changes in diffusion when water saturated acetylated cell walls expand at a constant water content. The most substantial changes in diffusion occur where the additional acetylation displaces water, decreasing the moisture content for the cell wall. These findings elucidate the molecular mechanism through which acetylation affects secondary plant cell walls at atomic resolution.

## Graphical abstract

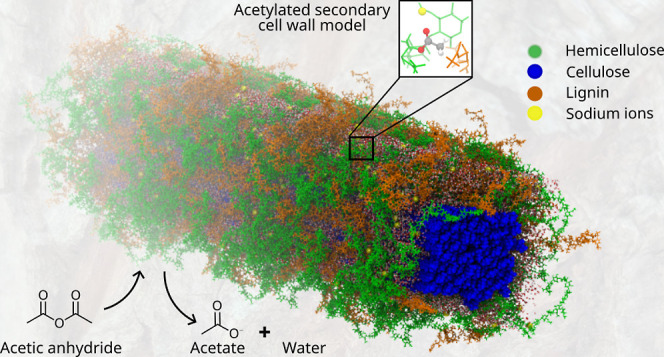

## Introduction

Wood is one of the oldest construction materials, with documented use 1.5 million years ago.^1^ Since then, wood product utilization has evolved gradually. Given its abundance as a natural resource, wood is currently used in several other industries beyond construction with an aggregate global consumption of 3.1 billion m^3^ in 2022.^2^ Wood consumption is expected to grow 37% by 2050 according to UN reports,^2,3^ driven by an anticipated increase in demand for wood in construction,^4,5^ biofuels,^6,7^ and other biomaterial industries.^8^ Greater utilization for this renewable resource can be facilitated by a nanoscale understanding of wood polymers and their interactions at all scales,^9–11^ with an eye toward improving its durability and longevity.

The largest contributor to the dry weight of woody biomass is the secondary plant cell wall. The secondary cell wall is a tightly interconnected assembly of lignin, hemicellulose, and cellulose polymers,^12–14^ which together strengthens the plant mechanically, and act as a barrier against microbial or fungal attack.^15,16^ Living plants have many approaches to keep pathogens at bay, but once these defenses are bypassed or the plant is processed industrially, environmental and fungal degradation processes will irreversibly degrade wood properties.^17^ Chemical and physical treatments can modify the underlying wood structure to aid in preservation, enhancing wood durability.^18–20^ One popular modification technique is acetylation, with ≈60,000 m^3^ of acetylated wood produced globally in a given year.^21^

Cell wall polymers within wood are acetylated commercially by reacting wood with acetic anhydride. Acetic anhydride reacts with exposed hydroxyl groups in the cell wall, replacing them with the acetyl group in a single reaction^22^ (Figure 1). Wood acetylation is particularly valuable in preventing brown rot decay, where iron and chelator-mediated Fenton (CMF) reagents released by fungi diffuse into the wood, creating hydroxyl radicals that depolymerize cell wall polymers.^23^ Wood acetylation inhibits brown rot decay,^24–26^ with slower CMF reagent diffusion proposed as the likely mechanism.^27,28^ Furthermore, acetylation also enhances the dimensional stability,^29^ increases wood hardness by 15% to 30%,^30^ and reduces moisture content and polysaccharide-water interactions.^31,32^ Thus, the improved durability and retained toughness of acetylated wood makes it an exceptional green construction material with CO_2_ negative life-cycle-analysis over full-life-cycle,^28^ and a highly attractive biomaterial for a burgeoning bioeconomy.

**Figure 1:**
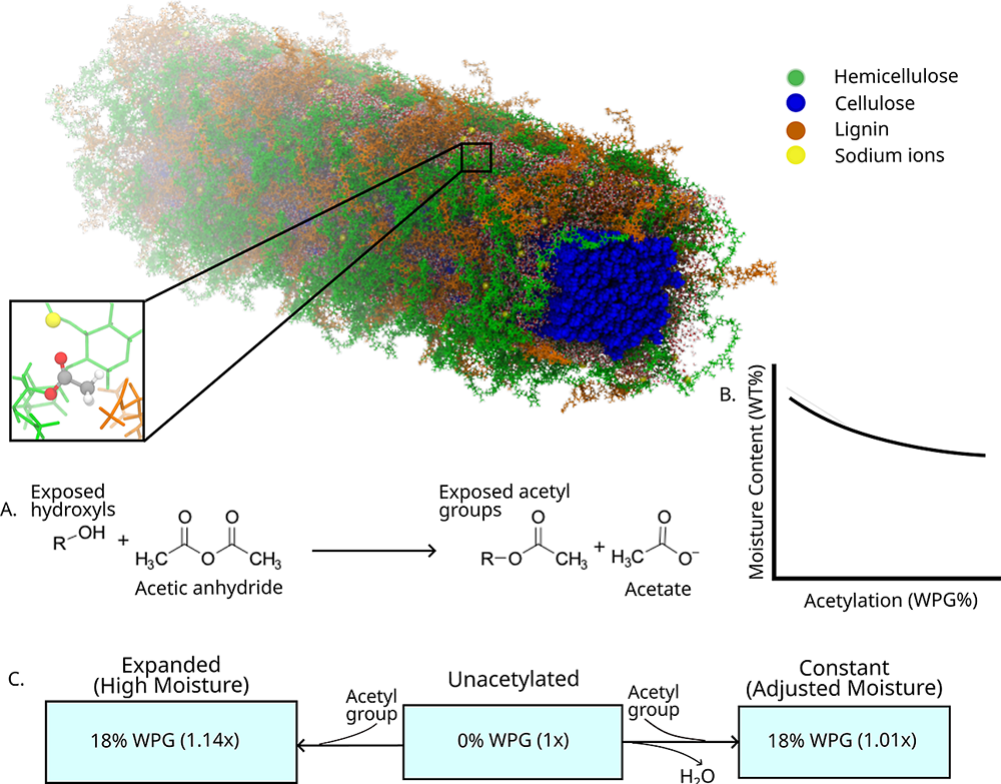
Schematic for the acetylated secondary plant cell wall model. The secondary plant cell wall model utilized here is a representative of hardwood species, starting with lignin (brown), hemicellulose (green), and cellulose (blue) solvated in 30% water weight (% WT) and 35.5 mM concentration of Na^+^. (A) The acetylation treatment involves the deprotonation of exposed hydroxyl groups by acetic anhydride to produce acetyl groups. (B) The moisture content of wood cell walls is reduced as the acetylation level increases (% weight-percent gain (WPG)).^32,35,38,39^ (C) Unacetylated cell wall model has been acetylated using two distinct mechanisms, (i) expanded wherein only the acetyl group is added with no other changes yielding an increased box volume and (ii) constant system where acetyl groups directly displace water molecules yielding a relatively consistent box volume.

While the impact of acetylation on increased durability is clear, the mechanisms at the molecular level that contribute to this durability is an active area of research with competing hypotheses.^27,33^ Hydration is clearly involved somehow, as increasing acetylation as measured by weight-percent gain (WPG) reduces the moisture content within plant cell walls^31,34^ (Figure 1), which had been previously hypothesized by prior X-ray fluorescence microscopy (XFM) measurements.^35^ Comparing the ion uptake and diffusion between acetylated^34^ and nonacetylated^36^ wood samples determined by XFM, the ion and biopolymer diffusion within the acetylated wood samples are slower but Fickian, as we would expect for simple diffusion. One proposed hypothesis to explain these trends is that the acetyl groups make specific interactions with ions that would retard diffusion, keeping the ions locked in close contact with the biopolymers rather than in larger dynamic water pockets. Another potential rationale for slower diffusion is that increased acetylation displaces water from the cell wall structure. A lower moisture content within secondary cell walls has been associated with reduced diffusion and structural transitions by both the experiment^36^ and simulation.^13,37^

The molecular level picture of a plant cell wall provided by molecular dynamics (MD) simulations (Figure 1) is well positioned to discriminate between these alternatives, with many examples in the literature where MD provides a molecular picture of the nanoscale interactions that control the cell wall structure or dynamics.^9,11,14,40–43^ By simulating both (1) a constant water content where acetylation takes place but the number of water molecules remains fixed, creating an expanded cell wall to check whether acetyl interactions are sufficient to slow diffusion, and (2) where acetylation directly replaces water molecules to create a cell wall with a constant size but decreasing hydration to test if reduced hydration is the primary driver of reduced diffusion. While diffusion is anticipated to reduce substantially upon acetylation,^34,44^ our simulations indicate that this only occurs when acetylation displaces water molecules filling in space between the cell wall polymers. Water pockets, alongside other quantities of interest such as relative affinities of ions to specific functional groups, have been measured directly from the simulations to appropriately quantify the structural and dynamical changes within secondary cell walls upon acetylation.

## Methods

To test the impact of acetylation on the structure and dynamics within plant cell walls, we have developed two sets of molecular simulation systems based on prior cell wall models used to probe ion diffusion.^13^ In both cases, we vary the degree of acetylation from a fully unacetylated control to a fully acetylated system, where every exposed hydroxyl on hemicellulose and lignin polymers has been acetylated. The unacetylated model starts with a 30% water weight moisture content. The “Expanded” system retains the 30% moisture content after acetylation, facilitating analysis of what happens when only acetylation takes place and the rest of the system is unperturbed and facilitates a direct comparison with unacetylated controls (1C). “Constant” system conversely has a constant volume upon acetylation, as acetyl groups directly displace water, reducing the water content to 19.4% once fully acetylated. In practice, the reduced water content of the “constant” system is much closer to the physical reality of acetylated wood.^38^ Each of these initial structures were simulated for 1 μs over multiple simulation replicates.

### Atomistic Plant Secondary Cell Wall Model Construction

There are many potential secondary cell wall models from multiple organisms, including those that agree well with solid-state NMR data.^43^ In principle, we could develop a new secondary cell wall model, if we so choose. However, because genome wide association studies indicate that there is not a single composition for woody biomass, but rather a composition distribution,^45,46^ we instead elect to start from a prior cell wall model used in the group^13^ to facilitate easier comparisons.

The molecular plant secondary cell wall model (Figure 1) reused the earlier reported 30% water weight (wt %) system from Sarkar et al.^13^ The cell wall composition in the model is 49.6% cellulose, 22.5% hemicellulose, and 27.5% lignin by dry weight.^13^ The cellulose polymers are arranged in four 18-chain cellulose *I*_β_ bundles with a degree of polymerization (DP) 40.^47^ The hemicellulose fraction is similarly modeled as an extended xylan molecule with DP 40 as well. Lignin is represented using a 20-mer model as proposed by Ralph et al.^48^ for hardwood, composed of 13 syringol units and 7 guaiacol units. The unacetylated model from Sarkar et al.^13^ starts with a density of ≈1.29 g/cm^3^ with box dimensions 91.0Å by 91.0Å by 210.9Å and approximately 190,000 atoms. Over the course of acetylation, the density of the expanded system reduces to ≈1.28 $\frac{g}{cm^{3}}$ at 18% WPG, while it increases to ≈1.30 $\frac{g}{cm^{3}}$ for a constant system at 18% WPG (Table S1)

At this stage, the model was acetylated by chemically modifying exposed hydroxyl groups on hemicellulose and lignin polymers. Prior studies suggest that the dense packing of cellulose chains within a secondary cell wall precludes substantial acetylation.^49^ Using a custom-patch and psfgen in Visual Molecular Dynamics (VMD),^50^ our acetylation procedure replaces the water-exposed hydroxyl functional group with an acetyl functional group. The script first identifies the atom indices for the hydroxyl functional group in lignin and hemicellulose, which are within 3, 6, or 12  Å distance from water. In addition, we have a fourth scenario, where all hydroxyl in lignin and hemicellulose are acetylated, corresponding to an 18% increase in dry weight. The precise WPG for each scenario, as well as the fraction of all total hydroxyl groups that are acetylated, are reported in Table 1. The acetylation degree for a specific model is measured via a % dry weight gain, which is quantified through eq 1 using the dry mass both before and after acetylation (*m*_pre_ and *m*_post_, respectively).

1 $$\%\;dry\;weight\;gain\left(WPG\right)=\frac{m_{post}-m_{pre}}{m_{pre}}\times 100$$

**Table 1:** Degree of Acetylation Based on Water-Exposed Hydroxyl Groups in Lignin and Hemicellulose^a^

degree of acetylation					
distance from water (Å)	0	3	6	12	∞
% hydroxyl groups acetylated	0	29	54	82	100
% dry weight gain (WPG %)	0	5	10	15	18

a The % dry weight gain is computed based on eq 1. Distance from water (or ∞) represents the molecular system where all hydroxyl functional groups from lignin and hemicellulose are acetylated. 18% dry weight gain is the maximum acetylation possible in the model molecular system based on the number of exposed hydroxyl groups in the original model.

At this stage, we have five independent degrees of acetylation that we intend to run but we will do so with two different degrees of hydration. The “expanded” system is the default, where the amount of water is unchanged by increasing acetylation. Because acetylation increases the number of atoms present, the system naturally expands at constant pressure, creating a measurable volume change (Δ*V*). The “constant” system removes water upon acetylation to maintain a constant volume, thereby reducing the moisture content to that seen in acetylated cell walls.^51^ Table 2 lists the system size with the adjusted water weight, or the number of water molecules removed for each % dry weight gain after acetylation for the “constant” systems. As the dry weight increases, using eq 2, a larger number of water molecules are removed from the system, such that the 18 WPG % acetylated cell wall contains about 19% water weight (Table 2).

2 $$N_{water\;removed}=\frac{\rho_{H_{2}O}\cdot N_{A}\cdot \Delta V}{{MW}_{H_{2}O}}$$

**Table 2:** Evaluating the Reduced Moisture Content Necessary to Generate the Constant Systems with Constant Volume across the Degrees of Acetylation, Achieved by Reducing Water Content Derived from eq 2

relating the constant volume systems with an equilibrium moisture content					
% dry weight gain (WPG %)	0	5	10	15	18
water weight % (WT %)	30	27.2	24.5	21.4	19.4
*N*_water removed_		2183	4196	6613	8171
molecular system size	190,803	190,219	189,050	187,764	186,780

The exact number of waters removed in the constant system (*N*_water removed_) is determined via eq 2. This depends on the change in the volume experienced by the expanded system (Δ*V*), and the density of water ρH_2_O, Avogadro’s constant *N*_A_, and the molecular weight of water ( $MW_{H_{2}O}=18.02$ g/mol).

Ionization by either monovalent (Na^+^) or trivalent (Fe^3+^) cation species was carried out to neutralize the system. Based on the number of anionic carboxylates present in the hemicellulose model, this worked out to be a 150 Na^+^ ions or 50 Fe^3+^ ions. This is approximately a 35.5 mM cation concentration for the monovalent cation and 11.8 mM for the trivalent cation.

### Molecular Dynamics Simulations

As in prior simulations, we use the CHARMM36 force field for lignin^52^ and carbohydrates^53–56^ to describe the essential cell wall biopolymers lignin, hemicellulose, and cellulose, respectively. However, acetylated lignin and hemicellulose were not previously parametrized. We used the CHARMM general force field (CGenFF)^57,58^ to develop models for acetyl-patched monomers to fill in missing parameters identified by Force Field Toolkit (ffTK) plugin in VMD^59^ (Figure S1). Counterion species (Na^+^ and Fe^3+^) were added using the solvate command in VMD to neutralize the −150 charge from the cell wall polymers. These parameters are included within the data archive available in Zenodo (https://zenodo.org/doi/10.5281/zenodo.14232466).^60^

Independent microsecond long equilibrium molecular dynamics (MD) simulations were performed for different acetylation degrees (0–18%), for monovalent (Na^+^) and trivalent (Fe^3+^) cation species. Prior to a production simulation with the GPU-resident integrator in NAMD versions 3.0a9 through 3.0b6,^61^ the systems were briefly minimized using NAMD 2.14 to arrive at a starting state for dynamics. Initial velocities were drawn from the Maxwell–Boltzmann distribution rather than using a long temperature ramp prior to dynamics. Each system was simulated for 1000 ns to allow for internal diffusion and evaluate structural rearrangements in the cell wall microenvironment. Following the conventions for the CHARMM36 force field, short-range electrostatics and van der Waals terms were cutoff at 12  Å with a 10 Å switch and the TIP3P water model.^62^ While other 4- or 5-point water models may be more accurate, these are not validated with the carbohydrate or lignin force fields because their parametrization explicitly assumes a 3-point water model with the same charge distribution as TIP3.^52–56^ Long-range electrostatics was handled through the particle mesh Ewald method^63,64^ with a 1.2  Å grid spacing. Each integration time step was set to 2 fs, with bond lengths to hydrogen restrained.^65^ A Langevin thermostat^66,67^ was used to maintain a 300 K temperature using a 5 ps^–1^ damping coefficient applied to all heavy atoms. Constant pressure was maintained at 1 atm via a semi-isotropic Langevin barostat,^68^ where the dimensions perpendicular to the cellulose fibril axis varied together.

### Analysis

The analysis of our trajectories was enabled by scripts written through a python-enabled version of VMD 1.9.4a58.^50^ The analysis scripts are available via Zenodo^60^ and use the NumPy,^69^ SciPy,^70^ and matplotlib^71^ libraries to facilitate analysis. We quantify the diffusion coefficients of different components in the cell wall microenvironment, intermolecular contacts between cell wall polymers and cationic species, and the radial distribution function (RDF) to further quantify contacts and to provide an independent estimate for relative binding free energies. The first 50 ns of the 1000 ns trajectory data have been disregarded for each analysis, to account for systems re-equilibrating upon acetylation (Figure S2). The trajectory frames are analyzed and saved every 0.2 ps during the analysis process.

### Diffusion Coefficient Determination

Diffusion coefficients in molecular simulation trajectories are quantified using the Einstein’s relation.^72^ The trajectories are aligned to the centrally located cellulose microfibril, eliminating drift based on the thermostat fluctuations and the initial velocities during system equilibration. The error bars for the diffusion coefficients visualize a standard error, obtained by slicing the trajectory into 19 50 ns segments and quantifying the standard error across these segments. However, as different cell wall components move independently, we find that different approaches are required to determine accurate diffusion coefficients from the independent samples.

#### Diffusion Coefficient of H_2_O

The diffusion coefficient of water is derived from the linear fit between mean squared displacement and time, as there are many water molecules present in the molecular simulation system, and the mean squared displacement grows linearly with time (Figure S4). The squared displacements of water molecules are computed by analyzing the unwrapped trajectory via a displacement-based unwrapping scheme^73^ for each acetylation index, where the change in the displacement of the water molecule is calculated relative to the first simulation frame after equilibration. These squared displacements are, thereafter, averaged to quantify the mean-squared displacement (MSD), as shown in eq 3, wherein *t*_ref_ refers to the time point at 50 ns.

3 $$MSD\left(t\right)=\left\langle {\left(r\left(t\right)-r\left(t_{ref}\right)\right)}^{2}\right\rangle$$

From the mean-squared displacement as a function of time, a diffusion estimate can be readily computed from the slope of the MSD vs time series for water

4 $$D\left(t\right)=\frac{MSD\left(t\right)}{6t}$$

#### Diffusion Coefficient of Other Plant Secondary Cell Wall Components

For the remaining components, cell wall biopolymers and ions, the diffusion coefficients were quantified by incorporating a sliding window (Δ*t* = 50 ns). Unlike water, where each molecule can move independently from the others, the tight network of interactions between biopolymers means that diffusion is coordinated between components. This reduces the number of independent samples, and so eq 4 can be nonlinear with the limited sampling, as observed in Figure S4. Therefore, the long trajectories for the components are analyzed over a sliding window of 50 ns trajectory blocks to calculate the diffusion coefficient for a fixed time interval Δ*t*

5 $$D\left(\Delta t\right)=\frac{MSD\left(\Delta t\right)}{6\Delta t}$$

This framework gives enough independent samples from a microsecond simulation to give a reliable diffusion estimate. As shown in Figure S3, substantially shorter lag times produce estimates that are based more on short-time thermal motion, while substantially longer lag times when calculating diffusion reduce the number of independent samples so much that the estimates become unreliable. In both eqs 3 and 5, a factor of 6 in the denominator indicates diffusion in three-dimensional Cartesian coordinates.

### Atom Interactions

#### Atom-Pair Contacts

To quantify interpolymer contacts over time, we evaluate eq 6 over all pairs of atoms within components *i* and *j* from the elements of interest.

6 $$C_{ij}=\sum_{ij}{\left[1+e^{5\left(d_{ij}-5Å\right)}\right]}^{-1}$$

Unlike the diffusion calculation, where the trajectory is unwrapped prior to analysis, the system is wrapped using the fastpbc wrap command available within self-compiled VMD 1.9.4a58. We utilize a smooth cutoff using the switching function (eq 6) around 5 °A to evaluate contacts between heavy atoms, with closer contacts being weighted more heavily than distant ones, which has been used in prior research to analyze intermolecular contacts in plant cell walls and related biopolymers^13,41,74–76^ (Figure S6). Because the biopolymers extend beyond the periodic box dimensions, we leverage the KDtree periodic distance implementation within SciPy^70^ rather than the VMD built-in contact calculation to account for contacts across the periodic boundaries of the molecular simulation system when measuring molecular distances. This approach is analogous to prior analysis for the unacetylated system.^13^

Intermolecular pairwise interactions between functional groups (acetyl, carboxyl, and hydroxyl) and Na^+^ ions are, thereafter, normalized by a functional group abundance to derive Δ*G* values for each interaction relative to the carboxyl groups (Table 3). This approach is grounded in statistical mechanics principles established by Chandler,^77^ wherein number of direct contacts or integrated RDF values are related to Helmholtz free energy through the following equation

7 $$w\left(r\right)=-k_{B}T\;\ln g\left(r\right)$$

Because we are operating at constant pressure rather than constant volume, the Helmholtz free energy becomes the Gibbs free energy instead. Thus, the interaction probability with a specific functional group (*P*_s_) when compared with the interaction probability with a carboxyl group (*P*_c_) can be used to estimate free energy differences for an ion to interact with specific moieties, as shown in eq 8.

8 $$\Delta G=\Delta G_{group}-\Delta G_{carboxyl}$$

9 $$=-RT\;\ln P_{s}-\left(-RT\;\ln P_{c}\right)$$

10 $$=-RT\;\ln\left(\frac{P_{s}}{P_{c}}\right)$$

**Table 3:** Relative Binding Free Energies Table Δ*G* Values for Interactions between Na^+^ Ions and Acetyl, Carboxyl, and Hydroxyl Groups in the Constant System, at Varying Degrees of Acetylation^a^

functional group	quantification	0% WPG	5% WPG	10% WPG	15% WPG	18% WPG
acetyl group	Δ*G*_atompair_ (kcal/mol)		1.18 ± 0.20	1.22 ± 0.18	1.25 ± 0.16	1.37 ± 0.14
	Δ*G*_RDF_ (kcal/mol)		1.24 ± 0.07	1.24 ± 0.08	1.33 ± 0.07	1.45 ± 0.06
carboxyl group	Δ*G*_atompair_ (kcal/mol)	0.00	0.00	0.00	0.00	0.00
	Δ*G*_RDF_ (kcal/mol)	0.00	0.00	0.00	0.00	0.00
hydroxyl group	Δ*G*_atompair_ (kcal/mol)	1.72 ± 0.16	1.40 ± 0.19	1.47 ± 0.19	1.38 ± 0.21	
	Δ*G*_RDF_ (kcal/mol)	2.26 ± 0.07	1.81 ± 0.07	1.91 ± 0.09	1.78 ± 0.14	

a We can estimate the free energy two ways: either from the relative population based on the number of contacts directly (Δ*G*_atom pair_), or from the relative integrated RDF values up to the first minimum (Δ*G*_RDF_).

Standard error for Δ*G* was derived using eq 11, wherein σ_s_ and σ_c_ are the standard errors of interaction probability with the selected group and carboxyl group, respectively. To determine a standard error, the trajectory was divided into 20 equal parts to enable the quantification of summary statistics such standard error based on the variance observed across these trajectory fragments.

11 $$\sigma_{\Delta G}=RT\cdot \sqrt{{\left(\frac{\sigma_{s}}{P_{s}}\right)}^{2}+{\left(\frac{\sigma_{c}}{P_{c}}\right)}^{2}}$$

Eq 1 provides an example Δ*G* standard error derivation for acetyl interactions at 15% WPG.

#### Radial Pair Distribution Function

The radial distribution function (RDF) defines the probability of finding a particle at a specific distance (*r*) from a reference atom. We use this formalism to compute the probability distribution of cations within 10 Å of carboxyl, acetyl, and hydroxyl functional groups. The built-in radial pair distribution function (RDF) within VMD 1.9.4a58 ^50^ is used to quantify the distance-based probability, from the nearest neighbor list interactions computed for each frame of the equilibrium trajectory. The integral of the RDF is related to the number of interaction ions have with specific functional groups (Table. S4). The trajectory was divided into 20 equal chunks to enable the quantification of summary statistics such standard error based on the variance observed across these chunks. Thus, the integrated RDF can be turned into a probability of interacting with a specific functional group, normalizing again to the number of specific functional groups in our simulation system. This allows us to determine the free energy differences again directly from these probabilities (eqs 8, 11), providing a check on our contact-based metrics. Because we see that ion-carboxyl group interactions are the most likely based on the Δ*G* values, the free energy of associating to a carboxyl is used as a reference state (Table 3).

#### Dwell Time Analysis

From the pair–pair interactions described above, we can also determine a dwell time for how long a specific interaction between the cations (Na^+^ and Fe^3+^) and select functional groups in lignin and hemicellulose (carboxyl (Figure 5), acetyl (Figure S15), and hydroxyl (Figure S16)) was present. A molecular simulation trajectory makes this straightforward as we are tracking across trajectory frames for how long a pairwise interaction lasts before breaking. The python implementation depends on evaluating if a specific contact comes within 6 Å, and then evaluating for how many consecutive frames this is true. Realizing that most interactions are short, we report the time-weighted cumulative distribution for these interactions to better represent how long a specific interaction observed in a simulation will last before breaking.

#### Water Pocket Quantification

The dynamics and structure of larger water pockets are also evaluated through VMD^50^ scripts. We define bulk water pockets by water molecules that are at least 5Å away from any cell wall biopolymer atoms and, therefore, are not involved in hydrogen bond interactions with the polymers.^78^ By tracking the number of water molecules that meet these criteria during the trajectories, we can identify changes in the water structure over time, balancing between bulk-like waters and water molecules bound up in directly interacting with the biopolymers.

## Results

The molecular picture of dynamics and structure offered by an atomic MD model for a plant secondary cell wall with varying degrees of acetylation can quantitatively test the hypotheses from the introduction, testing the mechanism of action for slower diffusion induced by increasing acetylation.^34,44^ We take advantage of the atomic resolution offered by MD to also quantify specific interactions and dynamical quantities such as ion-interaction times within the dense biopolymer mesh formed within a secondary cell wall and track the time evolution of interactions broadly between biomass components.

### Molecular Diffusion within Acetylated Cell Walls

Diffusion is the primary experimental metric that we are trying to recapitulate, and the molecular diffusion coefficients for different components of the plant secondary cell wall model are shown in Figure 2. The large cell wall biopolymers are minimally impacted by acetylation or ion content, with a very slow diffusion of the large cellulose fibril used to align the trajectory. Hemicellulose and lignin densely packaged around the cellulose diffuse with coefficients in the range of 2–6 × 10^–9^ cm^2^/s, with the slowest diffusion occurring for the least solvated structures, consistent with what has been seen previously.^13^ Unlike Sarkar et al.,^13^ which covered a larger span of moisture content, no glass transition is observed for hemicellulose between 0 and 18% WPG (Figure S5). Polymer acetylation increases the polymer molecular weight, which would be expected to decrease diffusion based on prior biopolymer diffusion measurements.^79^ These measures were evaluated across three repeats for a simulation of 18% WPG in the expanded system, with minimal variance noted, raising the confidence of our measurements across all other simulations (Table S2).

**Figure 2:**
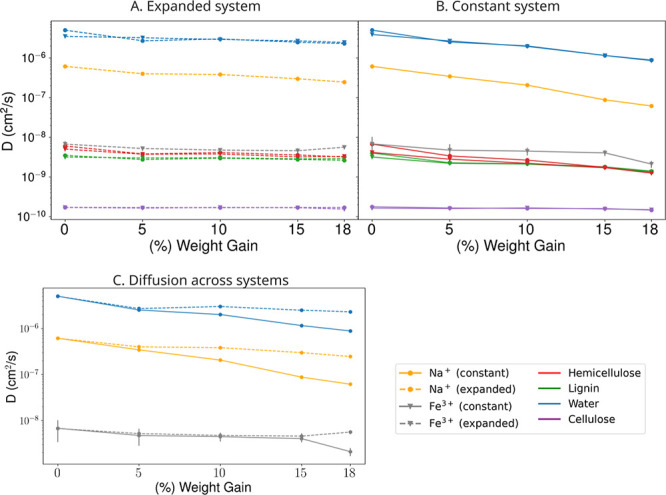
Quantifying diffusion coefficient for the secondary cell wall polymers and components across varying acetylation levels in the “expanded” and “constant” systems. Both (A) Expanded (dashed lines) and (B) constant (solid lines) systems have been simulated for 1000 ns across the five acetylation degrees, with mean diffusion coefficients for lignin (green), hemicellulose (red), cellulose (purple), cations (Na^+^-yellow, Fe^3+^-gray), and water (blue) visualized. Components within the systems neutralized by Na^+^ cations are denoted by circular markers while polymers within the system neutralized by Fe^3+^ are denoted by triangular markers. (C) A direct comparison between the evolution of diffusion coefficients for the cations (Na^+^ and Fe^3+^) and water in the expanded and constant system over the course of acetylation is depicted. Tabulated values are presented in Table S3.

The low impact of acetylation on diffusive behavior with a high water content (expanded system) is consistent throughout our analysis of Figure 2, where monovalent cation diffusion (Na^+^) is 2.5 × 10^–7^ cm^2^/s for a fully acetylated cell wall model, which is only 2.4*x* slower than 6.1 × 10^–7^ cm^2^/s for an unacetylated simulation. By contrast, when water is removed upon acetylation, the equivalent diffusion coefficient is approximately 6.2 × 10^–8^ cm^2^/s, an order of magnitude slower. Fe^3+^ diffuses approximately 10× more slowly, with measured diffusion coefficients of approximately 5 × 10^–9^ cm^2^/s. The Fe^3+^ diffusion coefficient is similar to the diffusion rates of lignin and hemicellulose at all levels of acetylation, which is much slower than the measured diffusion for Na^+^.

We observe that at reduced moisture levels present in the constant system the diffusion coefficients for Na^+^ and water reduce by almost an order of magnitude compared with the unacetylated control (Figure 2B). By contrast, the diffusion change upon acetylation for the expanded system is much smaller, decreasing by only 50% with the same change in acetylation (Figure 2A). The pattern is consistent for both Na^+^ and water, with Na^+^ trailing diffusion of water by a consistent factor of 8–10*x*. Naturally, a Na^+^ ion is heavier than water, so some slower diffusion is expected. However, the molecular weight difference is too small to account for the difference between water and Na^+^ diffusion. Instead, interactions between the cell wall polymers and cations are likely slowing ion diffusion, as has been noted previously for unacetylated systems.^13,36^

### Intermolecular Interactions between Acetylated Cell Wall Components

The interactions that slow diffusion are abundant and readily quantifiable within our molecular simulation system (Figure 3). Cell wall biopolymers interact with each other to a greater extent as the level of acetylation increases (Figure 3B). These interpolymer interactions in the constant system with reduced moisture grows faster compared to the expanded system. This would support the idea that acetylation primarily displaces water molecules and that it reduces the distance of the closest approach between biopolymers. The effect size is the largest for lignin-hemicellulose interactions, where the acetyl groups can interact between the polymers.

**Figure 3:**
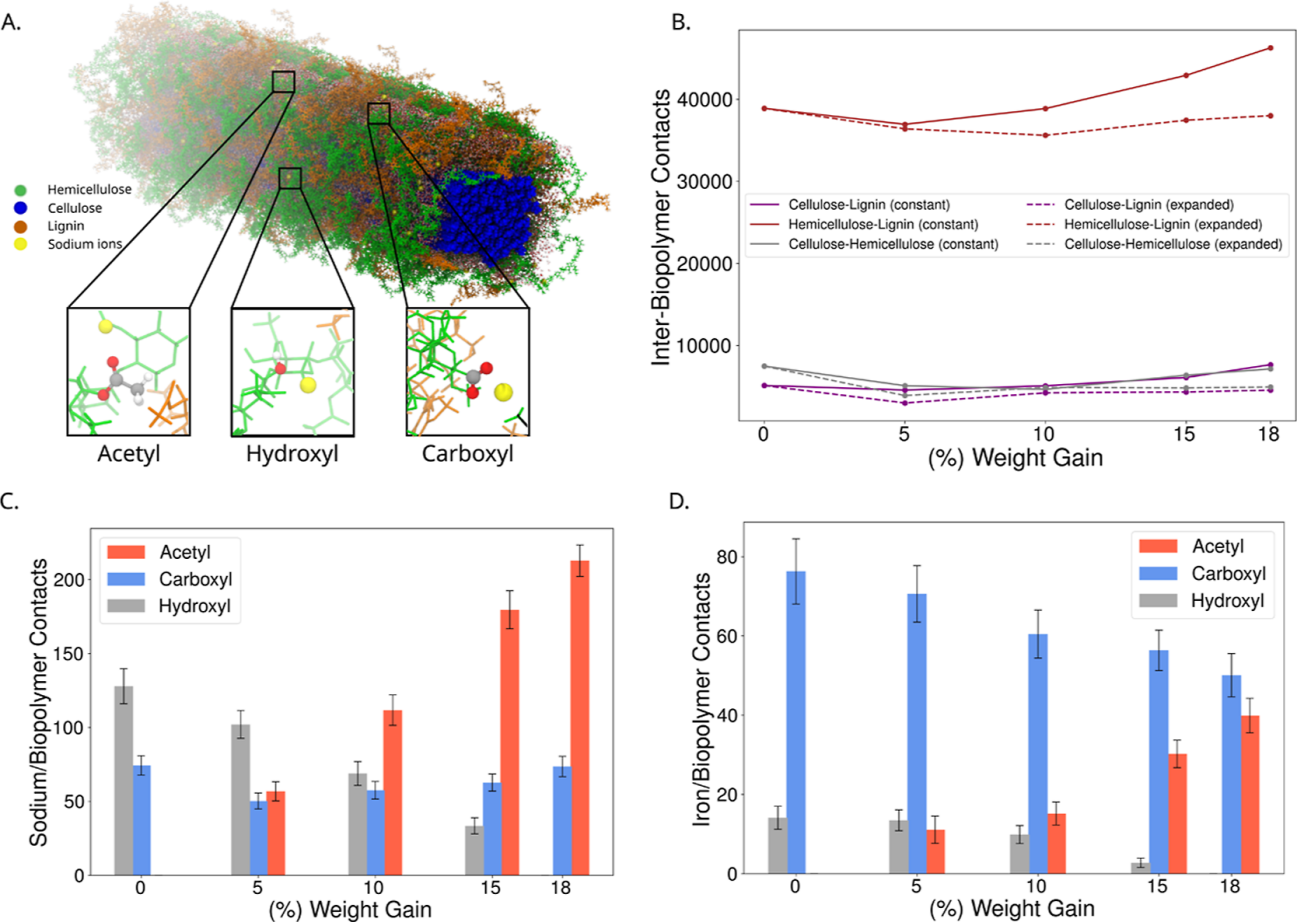
Specific interactions between ions and moieties on plant cell wall biopolymers. (A) Within our simulation, we can identify cases where Na^+^ ions (yellow spheres) interact directly with acetyl groups, hydroxyl group, and carboxyl groups in hemicellulose (green) and lignin (orange). (B) The interaction between the three secondary cell wall polymers (lignin, hemicellulose, and cellulose) for each degree of acetylation are quantified using eq 6, whose functional form is shown in Figure S6. (C,D) The interactions for specific lignin and hemicellulose moieties with 150 Na^+^ (C) and 50 Fe^3+^ (D) ions for each of the different acetylation degrees are quantified using the same approach as (B). In panels C and D, we are measuring the contacts for the “constant” simulation set, with the equivalent measures for the “expanded” simulation presented in Figures S7 and S8. Standard error estimates for each condition are a result of subdividing the 1 μs trajectory, where the first 50 ns is not included in the analysis to determine the mean.

Beyond polymer–polymer interactions, the simulations can directly quantify cation interactions with the cell wall microenvironment, tracking both Na^+^ and Fe^3+^ interactions at molecular detail (Figure 3). The cations largely make interactions with polar moieties, such as hydroxyls, carboxyls, and the acetyl groups within the biopolymers, and examples of these interactions are readily findable within our trajectories (Figure 3A). Panels C and D in Figure 3 quantify the interaction shift that takes places in the constant size systems with increasing acetylation for Na^+^ (Figure 3C) or Fe^3+^ (Figure 3D), with the equivalent measures for the “expanded” simulation presented in Figures S7 and S8. Without acetylation, ions can only strongly interact with hydroxyl or carboxyl groups, either in approximately equal proportion (Na^+^), or heavily weighted toward the carboxyls (Fe^3+^). As the hydroxyl groups are replaced with acetyl groups in the acetylation process, the total interaction count increases, sometimes substantially. For instance, Na^+^ ions have nearly twice the contacts with acetyl groups in lignin or hemicellulose at 18% WPG than compared to hydroxyl groups at 0% WPG. Some of this increase is clearly due to the increased size for the acetyl group compared to the hydroxyl group. However, there are also clearly many ions near the acetyl groups. There are only 150 carboxyl groups in our cell wall model (Figure S9), which is comparable to the number of cations present in the system to neutralize it. Thus, each ion likely only has one carboxyl group coordinating it, and thus other interaction partners, such as water, acetyl, or hydroxyl groups, further coordinate the ions to complete their solvation shell. As ions can interact with multiple moieties simultaneously, Figure 3C,D shows more ion-biopolymer contacts than the total numbers of ions.

The multicomponent coordination for the ions is clarified further by plotting the radial distribution function (RDF, also called a pair–pair distribution function) to evaluate close contacts with the ions (Figures 4, S10–S12). Focusing on the interactions Na^+^ ions make with their surroundings, the RDF peak is nearly equally tall between carboxyl and acetyl groups, complementing the contact view from Figure 3, and highlighting the importance of acetyl–ion interactions with a metric that is less biased by the number of atoms involved. Whereas the peak for the carboxyl group is consistently between 3 and 5, regardless of the acetylation degree, the peak grows substantially for the acetyl groups as the number of acetylations increases. The strength of the acetyl–ion interaction clearly cannot compete with the strength of the electrostatic attraction between the positively charged cation and the negatively charged carboxyl group. Comparing the Na^+^ ion interactions with acetyl, carboxyl, and hydroxyl groups, the interactions with carboxyl groups are observed to have the smallest relative Gibbs free energy (Δ*G*) (Table 3), followed by acetyl and hydroxyl groups. This also suggests that the Na^+^ interactions with carboxyl groups are expected to have the lowest dissociation constant (*K*_d_), while a lower *K*_d_ for interactions with the acetyl group compared to hydroxyl groups is another factor contributing to the reduced diffusion observed after acetylation. Indeed, there are approximately 28 times more acetyl groups at 18% WPG compared to the relatively rare carboxyl groups. Therefore, even though each cation-acetyl group interaction is relatively weak, the abundance of acetyl groups (4098 at 18% WPG) creates a significant cumulative effect. Given that cations interact more strongly with acetyl groups than hydroxyl groups (Table 3), wood acetylation increases the potential for ions to be held up as they diffuse through the material. Indeed, there are by construction an equal number of acetyl groups in the fully acetylated system as there are hydroxyl groups in the unacetylated system; thus, the ∼5*x* change in the peak heights between hydroxyl and acetyl systems highlights this strengthened interaction.

**Figure 4:**
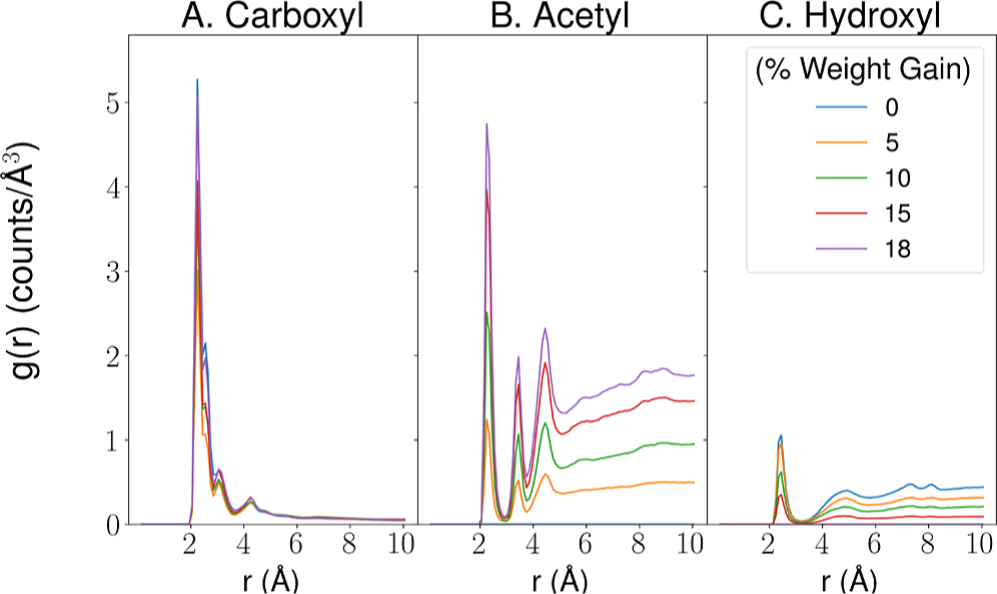
Radial Distribution Function (RDF) profile for interactions between Na^+^ ions and moieties on plant cell wall biopolymers in the constant system. RDF plots show the density of (A) carboxyl group, (B) acetyl group, and (C) hydroxyl group heavy atoms at distance *r*, within 10 Å of the Na^+^ ions in the constant size system. The densities have been tabulated at each acetylation degree using the built-in functionality of VMD^50^ to determine the RDF.

Perhaps the most interesting feature of Figure 4, as well as the RDFs for the other systems under study (Figures S10–S12) is the inherent structure to the RDF. Normally, subsequent peaks in an RDF are an indicator of a second or third shell of somewhat weaker interactions that are induced by long-range structures. However, the secondary peaks in the acetyl group RDFs result from the internal structure of an acetyl group (Figure S13). The primary peak is largely driven by the carbonyl-like oxygen that has both a relatively large partial negative charge and is very accessible to a cation. The second peak in the acetyl RDF comes from the ester carbon, and the third is a combination of the remaining oxygen and methyl carbons (Figure S13). In this way, the acetyl groups can cooperatively coordinate cations, even if the additional carbon and methyl groups contribute to molecular hydrophobicity.

Our focus to this point has been on the Na^+^ rather than on the Fe^3+^ that actually engages in Fenton reactions because the Fe^3+^ actually rarely interacts with anything other than the carboxyl groups. As seen in Figures S11 and S12, Fe^3+^ interacts most closely with carboxyl groups. Indeed, if one compares the interaction lifetimes for how long an ion remains bound, only a small fraction of Fe^3+^ ions are mobile at any time (Figure 5), as the electrostatic interactions tying the ions to carboxylates are quite strong. While the median time a Na^+^ ion remains bound to a carboxyl group is on the order of 10 ns, the median time for a Fe^3+^ ion to remain bound to the same carboxyl group is over 1000 ns, effectively the length of the simulation. This is only seen for Fe^3+^ interacting with the hemicellulose carboxyl groups, as hydroxyl or acetyl group dwell times (Figures S15 and S16) do not show a similar pattern, and may reflect limitations for how Fe^3+^ is handled within a fixed charge force field like CHARMM. Instead, the general trend is that interactions with carboxyl or acetyl groups get longer as acetylation increases. Our interpretation is that the reduced biopolymer diffusion with increasing acetylation slows the jostling that might dislodge an ion interaction, increasing the dwell time for an ion on the biopolymer. Ion interactions with hydroxyl groups appear to be less impacted by either hydration levels or acetylation, as might be expected given the stronger interactions with carboxyl and acetyl groups.

**Figure 5:**
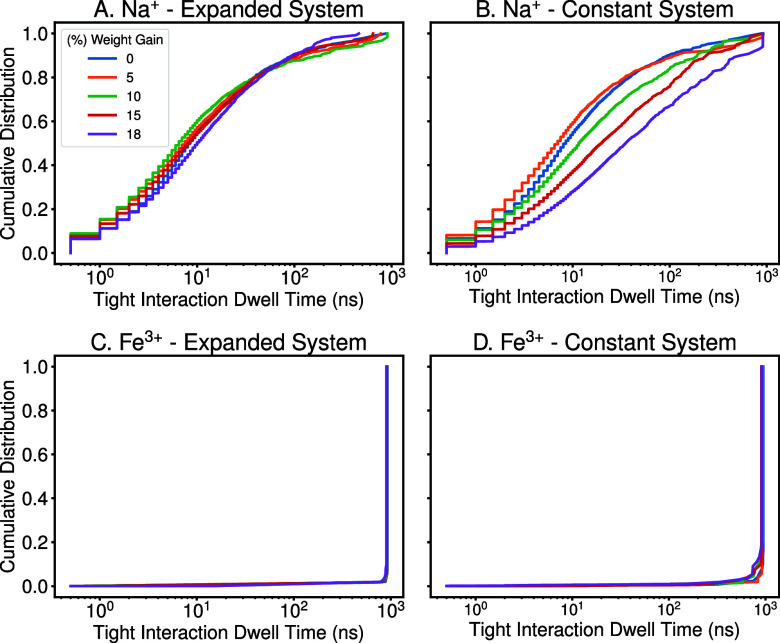
Dwell time analysis of cation interactions with carboxyl groups in “expanded” and “constant” systems at each acetylation degree. The interaction dwell time measures how long a specific ion remained bound to a particular carboxyl groups, showing the dwell time duration against the proportion of interactions exhibiting this behavior. Quantifications were performed at each acetylation degree within the following systems: (A) expanded system neutralized by Na^+^ ions, (B) constant system neutralized by Na^+^ ions, (C) expanded system neutralized by Fe^3+^ ions, and (D) constant system neutralized by Fe^3+^ ions. Most of the Fe^3+^ ions had interaction dwell times well beyond the simulation duration 1000 ns.

Indeed, the relative abundance of atomic interactions can be directly assigned into a relative Δ*G* value to quantify the relative interaction strengths (Table 3) through the application of eq 8. Carboxyl-ion interactions are the strongest, and by comparison acetyl group interactions are 1.18–1.45 kcal/mol weaker at binding to cations. Hydroxyl groups have weaker interactions still and are between 1.38 and 2.26  kcal/mol weaker at binding ions within the cell wall than carboxyl groups. The acetyl group binding affinities are more consistent between simulations than the hydroxyl groups, possibly a consequence of more interactions in general facilitating better statistical certainty, particularly as the reference are the rarer but stronger carboxyl interactions. As very few ions were uncoordinated by one of these three groups, the unbound population was very small and could not be used to reliably use the unbound state as a benchmark for other binding affinities.

### Solvent Structure in Acetylated Cell Walls

The overall change in the water structure within the cell wall upon acetylation is perhaps best quantified by visualizing the water pockets (Figure 6). Comparing end states from our simulation, we see that the water will often cluster together into the voids left by the cell wall polymers. The voids are visually much larger in the unacetylated cell walls compared with the fully acetylated structures (Figure 6A). Quantitatively, the removed water when acetylating the “constant” system is effectively all taken from these bulk water voids, as the bulk water fraction shrinks considerably as acetylation increases (Figure 6B). Indeed, the bulk water fraction is approximately 8× higher in unacetylated cell walls and is far in excess of the change in the hydration level alone, which only changes by approximately 50% (Table 2). Our interpretation is that with increasing acetylation, the amount of water bound up with the biopolymers makes up a greater and greater portion of all water molecules. Thus, the nanostructure of acetylated wood has much less room for solutes to diffuse, and very few water highways along which ions or other solutes could hitch a ride deeper into the secondary cell wall.

**Figure 6:**
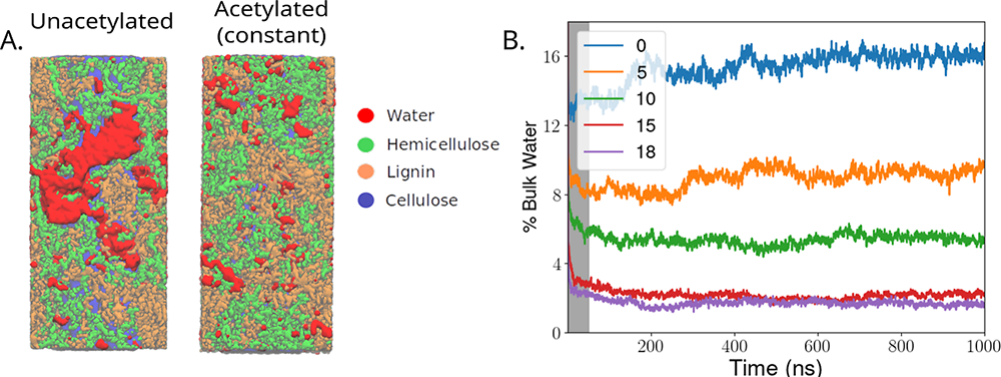
Water pockets across constant systems through different degrees of acetylation. (A) Within the simulated model with the “constant” simulation approach, either for the unacetylated system, or the 18%WPG. We identify water molecules in the bulk that are at least 5 Å away from cell wall biopolymers. The fraction and size of these bulk water pockets clearly is reduced as acetylation increases within the secondary cell wall. (B) The evolution of these pockets of bulk water over the 1000 ns is visualized using a time series at each acetylation degree. In this panel, we are again measuring the percent of the total water that is at least 5 Å away from any cell wall biopolymer atoms. As a reminder, the first 50 ns of the trajectory is considered equilibration, and is marked in gray.

## Discussion

Acetylation has been proposed to hinder fungal decay through reduced CMF reagent diffusion across the plant cell wall.^27^ Slower ionic diffusion and ion uptake for Fe^3+^ has been observed in acetylated cell walls compared to unacetylated controls.^34^ Zelinka et al.^34^ measured a reduction of ≈10–15× for the Fe^3+^ uptake after 48 h in acetylated wood at 20% WPG. Our simulations of fully acetylated secondary cell wall systems similarly show a ≈10× reduction in Na^+^ diffusion coefficients. This close qualitative agreement leaves little room for doubt that the models simulated here capture something real happening in acetylated wood.

However, there are caveats, as the cations and measurement techniques vary between this study and prior experiments.^34^ Nevertheless, capturing this change is a big step forward in understanding the impact of the plant cell wall structure on the macroscopic properties in wood. For instance, losing an order of magnitude in the diffusion coefficient for ions when the system is acetylated reduces the characteristic diffusion length L by a factor of between 3 and 4, as the distance a particle diffuses in a given time is proportional to the square root of the diffusion coefficient and time.[Fn fn1] Thus, the penetration over an hour of the solution uptake would be expected to be 3 times less for acetylated wood, at least for small monovalent cations. Concretely, assuming a diffusion coefficient of 10^–7^ cm^2^/s, as is the case for Na^+^ in acetylated wood in our study (Figure 2), the ion would penetrate approximately 0.4 mm after 1 h of uptake, compared with 1.2 mm for unacetylated wood. Iron, which already diffuses much more slowly due to the strong interaction with the carboxyl groups on hemicellulose, is further slowed as the fraction of bulk water is reduced with increasing acetylation (Figure 6).

From the outset, there were two hypotheses to investigate around how diffusion is slowed by acetylation: (1) that acetyl groups directly interact with small-molecule solutes or (2) that the reduction in hydration results in slower diffusion. The diffusion measures in our two hypothetical cell walls clearly indicate that there is some merit to both hypotheses, as the diffusion went down with acetylation regardless of how much water was present (Figure 2). Ion interactions with cell wall biopolymers in Figures 3 and 4 indicate that Na^+^ ions interact more readily with the acetyl group than the hydroxyl group. This leads to an increased interaction dwell time as the cell wall is further acetylated and supports the hypothesis that acetyl–ion interactions hinder ion diffusion. Figure S13 highlights that the carbonyl-like oxygen on the acetyl group with an accessible partial negative charge is the leading candidate for this interaction with cations. In isolation, this would strongly suggest that the first hypothesis is correct, and we certainly see that diffusion slows with increasing acetylation under all conditions, as weaker hydroxyl interactions are replaced with stronger acetyl interactions (Table 3).

However, the change in diffusion is just much greater when hydration was reduced. Comparing the diffusion for the “constant” and “expanded” systems, the diffusion decreased with increasing acetylation by 10× and 2×, respectively (Figure 2C). Thus, while maximizing acetylation alone reduces diffusion by ∼2×, the reduction in the equilibrium moisture content from 30% to 19.4% (Table 2) contributes an extra ∼5× diffusion deceleration. This 5× change in ion diffusion had been noted previously for a similar change in the moisture content through simulation by Sarkar et al.^13^ The multiplicative effect of acetylation beyond what moisture content reduction alone would do is also influenced by the increased hemicellulose–lignin interactions facilitated by the additional bulk of the acetyl groups (Figure 3B). Compared with prior unacetylated wood studies,^13^ acetylation increases ion interactions with hemicellulose by ≈ 1.12 × and with lignin by ≈ 1.55× (Figure S14). Thus, the hydration mechanism in our view should be considered the primary mechanism by which diffusion and the uptake are slowed by wood acetylation.

Additionally, Figure S5 suggests that no glass transition is observed for hemicellulose across the degrees of acetylation. This is in line with Sarkar et al.,^13^ which observes glass transition between equilibrium moisture contents of 10–15% (WT). The simulated acetylation models, even at 18% WPG retain 19.4% WT (Table 2). This is still above the moisture-induced glass transition, suggesting that more moisture would need to be lost to observe a glass transition in these acetylated simulations. It is important to realize that we started from a moisture level that would be expected for living tissues rather than dried wood and, so, we would expect the reduced moisture content induced by chemical acetylation to trigger glass transitions if the initial moisture content would be lower.

A prior work has proposed that hemicellulose acetylation rather than lignin acetylation was the dominant biopolymer influencing diffusion in secondary cell walls.^27^ The proposed mechanism for the role of hemicellulose has been hypothesized to be that the hemicellulose and amorphous cellulose are prevented from passing through their moisture-induced glass transition due to acetylation.^27^ Experimental acetylation involves exposure to acetic anhydride and the accessibility to the acetic anhydride reagent is significantly greater in lignin compared to hemicellulose, yielding a relatively higher rate of acetylation in lignin.^80^ Lower WPG, therefore, display a greater extent of lignin modification; Rowell et al.^49^ estimated that at 8% WPG, approximately 80% of lignin hydroxides had been modified, while only 12% of the eventual hemicellulose modification has occurred. In our models, we do not directly account for this differential acetylation, as we are deciding what to acetylate based on water accessibility. Indeed, analysis indicates that our partially acetylated models have approximately equal numbers of acetyl groups on both lignin and hemicellulose, as we were using strict geometric criteria for adding acetyl groups. Because hemicellulose has many more hydroxyl groups than lignin does on a per-monomer basis, an equal number of acetyl groups means that more of the available lignin is modified overall.

While the lignin/hemicellulose acetylation fractions are roughly consistent with past experiments, there are other limitations inherent to the model. The secondary cell wall model here contains the three polymers (cellulose, hemicellulose, and lignin) in the approximate ratio found in a model hardwood (Table 4).^13,83^ It is not computationally feasible to simulate intact biomass where the polymers are at their native lengths, which can be up to several thousand monomers long.^81^ What is practical in our models is for more modest polymer lengths with up to 40 repeats along the long axis of the simulation. For lignin, this is likely fine, as the approximate molecular weights for lignin indicate that 20 monomers is a typical length in the Lignin Data set^82^ and is modeled as such in the simulated cell wall model. Glycans are a different story in hardwoods, with a degree of polymerization (DP) of ≈120 for xylan (hemicellulose) and ≈9300 for cellulose in the secondary cell wall of poplar.^81^ The DP values for the hemicellulose and cellulose are significantly lower in the simulated model than real cell walls. The short DP for the hemicellulose in the model may also play a role in preventing the observation of a moisture-induced glass transition.

**Table 4:** Degree of Polymerization for the Cell Wall Biopolymers in Our Cell Wall Model Adapted from Sarkar et Al.^13^ Compared to the Previous Experimental Literature Data^81,82^

degree of polymerization			
cell wall biopolymer	cellulose	lignin	hemicellulose(xylan)
experimental DP	9300 ^81^	20 ^82^	120 ^81^
simulation DP	40	20	40

Another potential limitation in the simulation may come down to how the relationship between the equilibrium moisture content and the degree of acetylation is modeled. Acetylation has been long known to reduce the water content.^32,35,38,39^ The simulation models utilized here capture this general trend, showing a ≈10% decrease in the equilibrium moisture content between unacetylated (30% WT) and fully acetylated wood (20% WT) (Figure S17). Experimental studies on forest products already in the value chain report a slightly more pronounced drop in the moisture content from 27% to 15% as wood is acetylated up to 18% WPG.^84^ Thus, while we replace the same amount of water with acetyl groups in the constant system as is relevant to the experiment, we would anticipate that the even lower hydration levels observed in experimental wood samples^34,35^ would further reduce diffusion below what we report here.

While the molecular models presented here are necessarily simpler than reality, they still hint at alternative approaches to modify the cation uptake and intercalation into plant cell walls. Considering that hydration is such a powerful handle to modify ionic diffusion, using bulkier chemistries rather than simply an acetic anhydride (Figure 1) would be expected to further drive down diffusion. Papadopoulos and Hill^85^ found that the equilibrium moisture content is proportional to WPG, and so bulkier and heavier groups that increase the WPG would displace more water per molecule added. Given the strong interactions between cations and carboxyl groups within plant cells (Figures 4, 5), genetic modifications to add additional carboxyl groups would be another handle with which to modify ion diffusion within forest products.

## Conclusion

The caveats to our molecular simulation results notwithstanding, we have a clear mechanism to explain why acetylation reduces cation diffusion within plant cell walls. Acetyl groups are better than hydroxyl groups at coordinating ions, and so, wood acetylation creates more points for ions to stick as they traverse internal water pockets within a secondary cell wall. This alone is responsible for a 2.4x reduction in the diffusion coefficient. However, the more significant driver of reduced diffusion is that acetylation displaces interstitial water molecules within a cell wall, dehydrating the polymer network. The effect of dehydration reduces the diffusion coefficient by a further factor of 5–8*x*. We also note that cations bind with a very long duration to anionic carboxy groups in hemicellulose. Together, these mechanistic insights provide the rational basis for alternative treatments that may lead to longer lasting and renewable building materials, such as by changing the chemistry to make the acetyl groups bulkier and thus displace more water or to genetically engineer trees themselves to amplify carboxylation in secondary cell walls.

## Supplementary Material



## Data Availability

The reduced directory structure that includes analysis scripts, inputs and selected raw outputs used for this publication is available from Zenodo (https://zenodo.org/doi/10.5281/zenodo.14232466). The complete directory structure is available upon request.

## Footnotes

a

$L=2\sqrt{Dt}$

## Notes

Density for the equilibrated systems, workflow depicting how acetylation was incorporated to the plant secondary cell wall model, box volume evolution over the course of simulation at the five degrees of acetylation within the constant and expanded system, diffusion coefficients for secondary cell wall components at varying offsets (Δ*T*), mean square displacement (MSD) time series plots, indicating evolution of MSD over the course of simulation, variance and standard error for diffusion coefficient values at 18% WPG over three simulation repeats, diffusion coefficients for cell wall components in all four systems, diffusion coefficients for cell wall polymers in the constant system with Na^+^ counterions on a linear scale, switching function that visualizes the quantification of atom pair interactions, calculation for Δ*G* error, interactions between Na^+^ ions and moieties on the plant cell wall polymers, interactions between Fe^3+^ ions and moieties on the plant cell wall polymers, evolution of functional groups’ quantities over the course of acetylation, RDF profiles for interactions between Na^+^ ions and plant cell wall biopolymer moieties in the expanded system, RDF profiles for interactions between Fe^3+^ ions and plant cell wall biopolymer moieties in the expanded system, RDF profiles for interactions between Fe^3+^ ions and plant cell wall biopolymer moieties in the constant system, RDF profiles for individual atoms in the acetyl group during their interaction with Na^+^ ions, RDF integrals for interactions between Na^+^ ions and the functional groups, evolution of inter polymer contacts over the five degrees of acetylation, dwell time analysis for cation interactions with acetyl groups across all four systems at each degree of acetylation, dwell time analysis for cation interactions with hydroxyl groups across all four systems at each degree of acetylation, and EMC change between the simulated model against previous literature data (PDF)

## ⊥

Molecular and Structural Biophysics, Laboratory of Chemical Physics, National Institute of Diabetes and Digestive and Kidney Diseases, National Institutes of Health, Bethesda, MD 20892, USA
