# Supplementary material for: Quantifying Chemically Modified Acetylation Induced Changes in the Plant Secondary Cell Wall Structure and Dynamics
Source: Biomacromolecules. 2025 Aug 21;26(9):5645–56. doi: 10.1021/acs.biomac.5c00391 (PMC12421508; doi:10.1021/acs.biomac.5c00391)
Supplement: Supplementary file 1 [file bm5c00391_si_001.pdf]

Supporting Information: Quantifying chemically modified acetylation induced changes in the plant secondary cell wall structure and dynamics

Murtaza Barkarar,† Daipayan Sarkar,‡,¶ Christopher G. Hunt,§ and Josh V. Vermaas*,‡,‖

†*Department of Biochemistry and Molecular Biology, Michigan State University, 612 Wilson Road, East Lansing, MI 48824, USA*

‡*MSU-DOE Plant Research Laboratory, Michigan State University, 612 Wilson Road, East Lansing, MI 48824, USA*

¶*Current address: Molecular and Structural Biophysics, Laboratory of Chemical Physics, National Institute of Diabetes and Digestive and Kidney Diseases, National Institutes of Health, Bethesda, MD 20892, USA*

§*USDA Forest Service, Forest Products Laboratory, Madison, WI 53726, USA*

‖*Deparment of Biochemistry and Molecular Biology, Michigan State University, 612 Wilson Road, East Lansing, MI 48824, USA*

E-mail: vermaasj@msu.edu

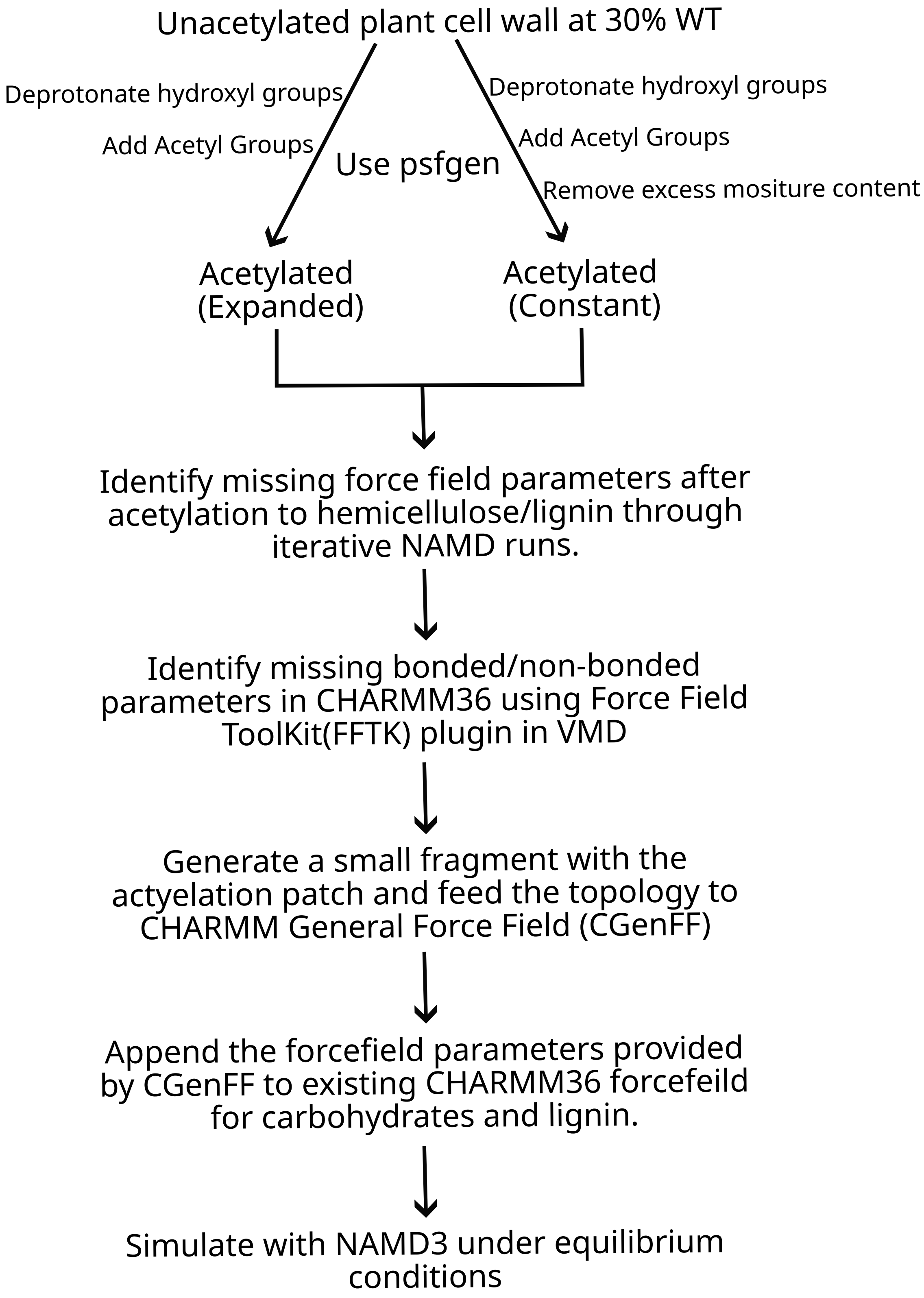

Figure S1: **Workflow describing how the system was generated**, including acetylation of the plant secondary cell wall model using psfgen and subsequent parametrization of the newly added bonds and angles through FFTK and CGenFF.

Table S1: Density ($\frac{g}{cm^3}$) in the expanded and constant system across the degrees of acetylation.

	0% WPG	5% WPG	10 % WPG	15% WPG	18% WPG
Expanded system	1.292 $\frac{g}{cm^3}$	1.290 $\frac{g}{cm^3}$	1.288 $\frac{g}{cm^3}$	1.284 $\frac{g}{cm^3}$	1.279 $\frac{g}{cm^3}$
Constant system	1.292 $\frac{g}{cm^3}$	1.304 $\frac{g}{cm^3}$	1.302 $\frac{g}{cm^3}$	1.307 $\frac{g}{cm^3}$	1.302 $\frac{g}{cm^3}$

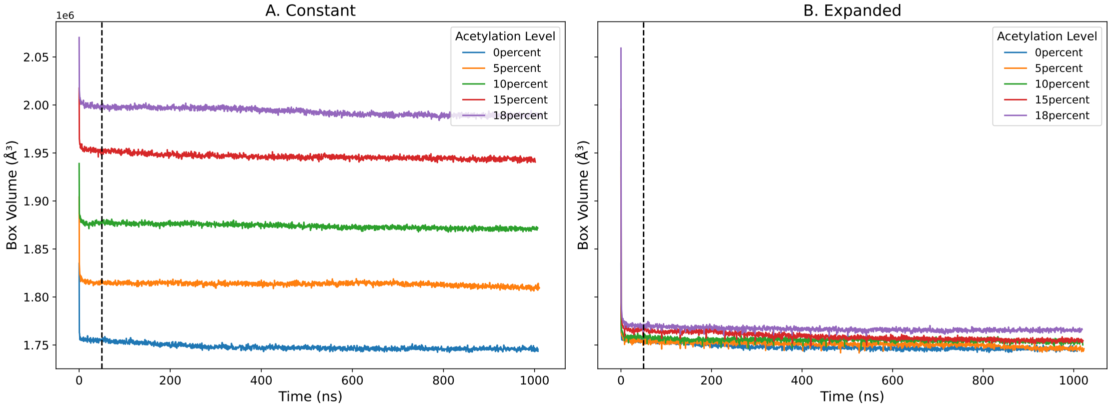

Figure S2: **Box Volume evolution over the course of simulation at different degrees of acetylation.** The Box volume evolution is visualized for all five degrees of acetylation in the A. Expanded system and B. Constant system. The vertical line at 50 ns indicates that box volume across all five systems has converged well before that.

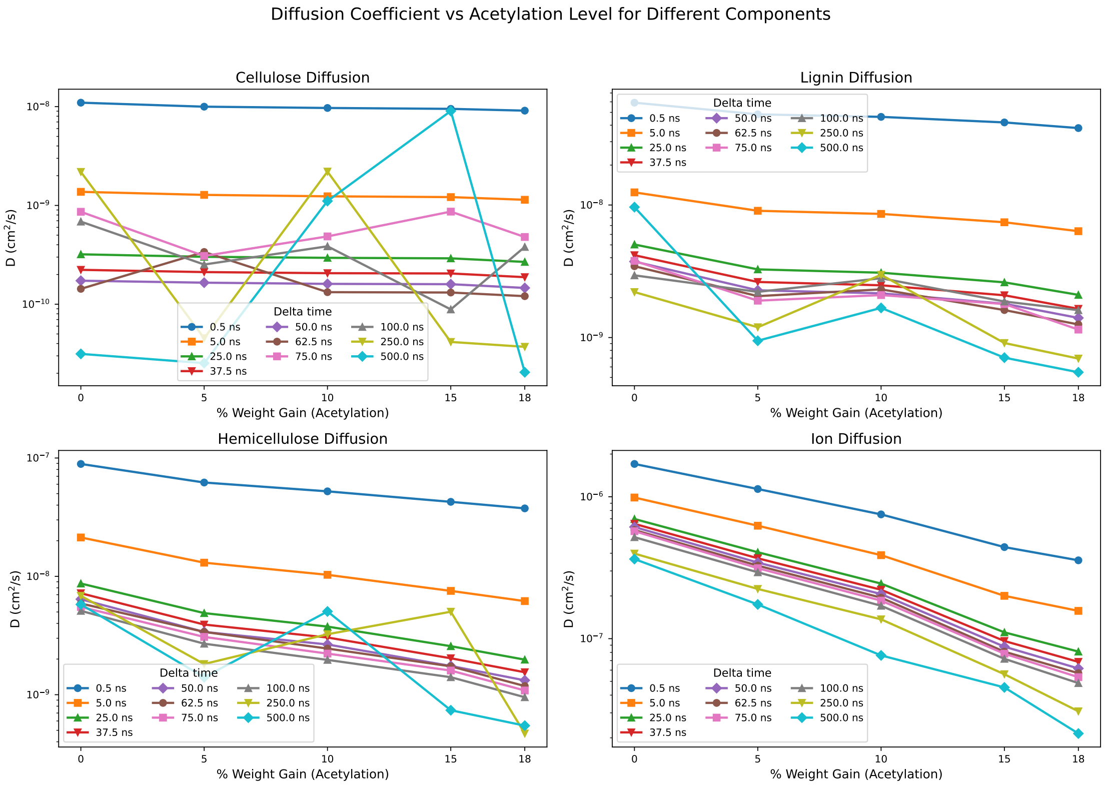

Figure S3: **Diffusion coefficients for lignin, cellulose, hemicellulose, and ions in constant system derived using different offsets.** Diffusion coefficients ($cm^2\,s^{-1}$) for A. Cellulose, B. Lignin, C. Hemicellulose, and D. Ions are plotted across the five degrees of acetylation. Ten ΔT values (0.5 ns, 5 ns, 25 ns, 37.5 ns, 50 ns, 62.5 ns, 75 ns, 100 ns, 250 ns, 500 ns) are utilized to identify the most appropriate ΔT to quantify diffusion coefficients for non-water components using Eq. 5.

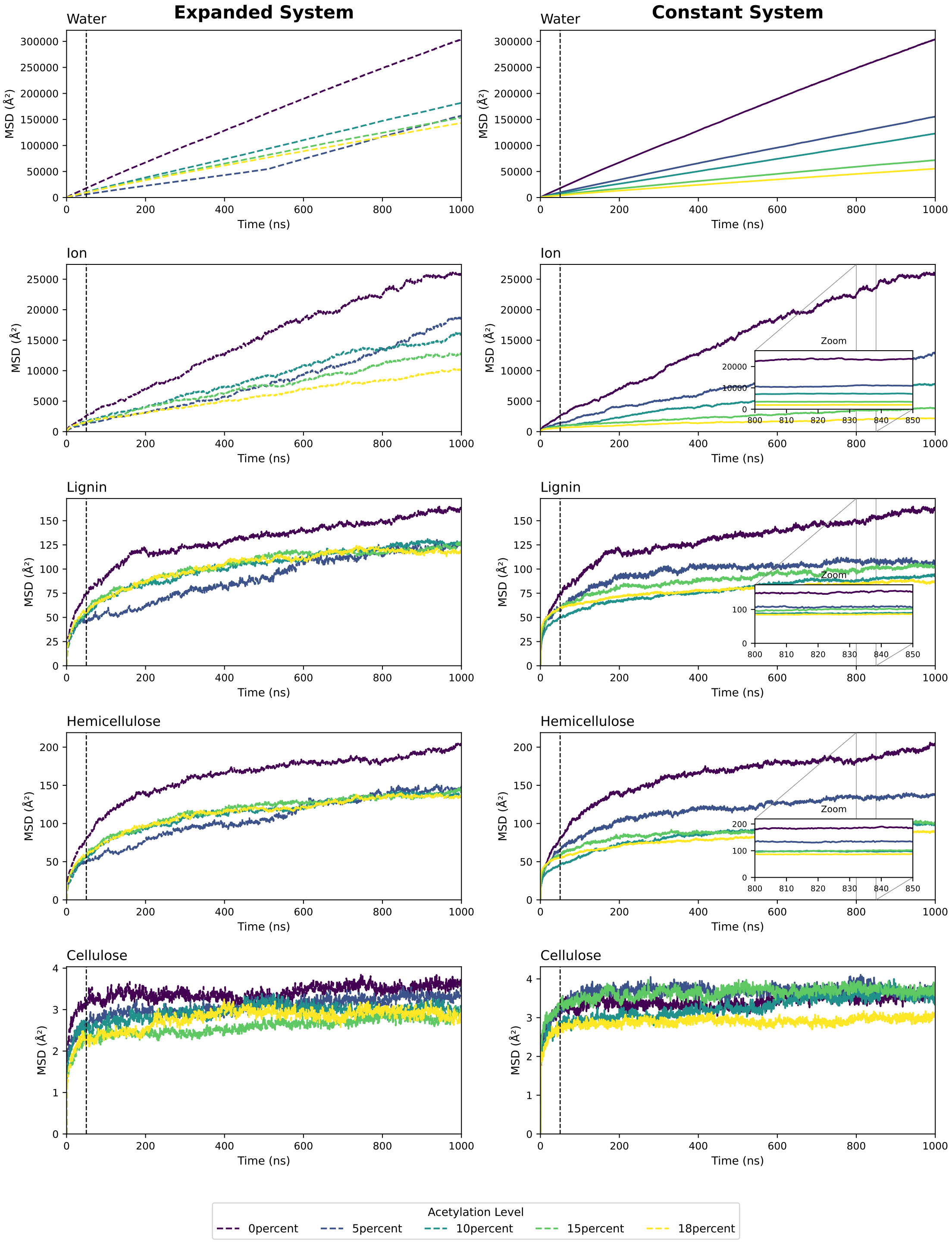

Figure S4: **Mean Square displacement time series plot** Mean square displacement (Å^2) for each of the cell wall component is plotted over the 1000 ns time series in the Expanded system and Constant system for each of the five components (Water, Na^+, Lignin, Hemicellulose and Cellulose), justifying the distinct approaches to quantify diffusion coefficients for water (Eq. 4) and the other cell wall components (Eq. 5). Mean square displacement for cellulose appears flat as the trajectory is aligned to cellulose, therefore mean squared displacement for all other components is measured with respect to cellulose.

Table S2: Variance and standard error for diffusion coefficients of cell wall components and ions at 18% WPG acetylation in expanded system over three independent simulations. Based off these results, we observe minimal variance for the diffusion coefficients across triplicates. As a result we choose to present the remainder of results for a single independent simulation, each a microsecond long.

Component	Cellulose	Hemicellulose	Lignin	Na^+	Water
Variance	5.54×10^{-25}	1.93×10^{-21}	2.46×10^{-22}	2.17×10^{-18}	5.54×10^{-25}
Standard error	3.51×10^{-13}	2.17×10^{-11}	7.40×10^{-12}	7.11×10^{-10}	2.16×10^{-9}

Table S3: **Diffusion coefficients of cell wall components and ions at 18% WPG acetylation**

Component	Na^+ Constant	Na^+ Expanded	Fe^{3+} Constant	Fe^{3+} Expanded
Cellulose	1.46×10^{-10}	1.70×10^{-10}	1.50×10^{-10}	1.55×10^{-10}
Hemicellulose	1.32×10^{-9}	3.20×10^{-9}	1.25×10^{-9}	3.31×10^{-9}
Lignin	1.41×10^{-9}	2.63×10^{-9}	1.34×10^{-9}	2.89×10^{-9}
Ion	6.17×10^{-8}	2.46×10^{-7}	2.11×10^{-9}	5.62×10^{-9}
Water	8.85×10^{-7}	2.31×10^{-6}	8.56×10^{-7}	2.46×10^{-6}

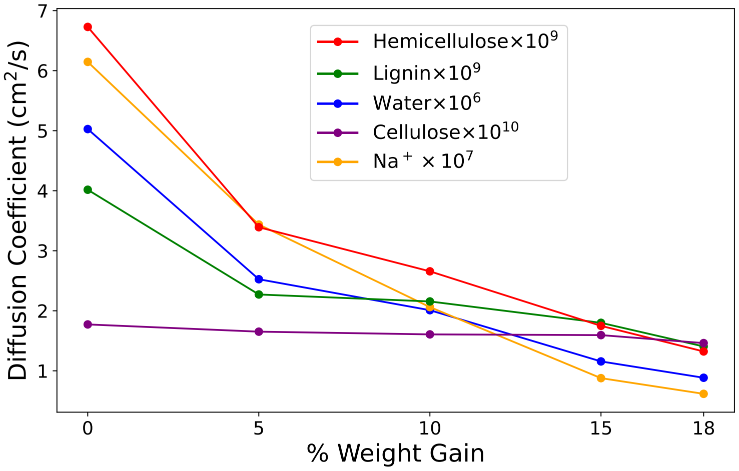

Figure S5: **Diffusion Coefficients for Na^+ ions, water and cell wall biopolymers in the constant system, depicted on a linear scale**. The linear scale suggests that the observed trend between diffusion coefficients of the components and the degree of acetylation is relatively continuous, indicating no glass transition.

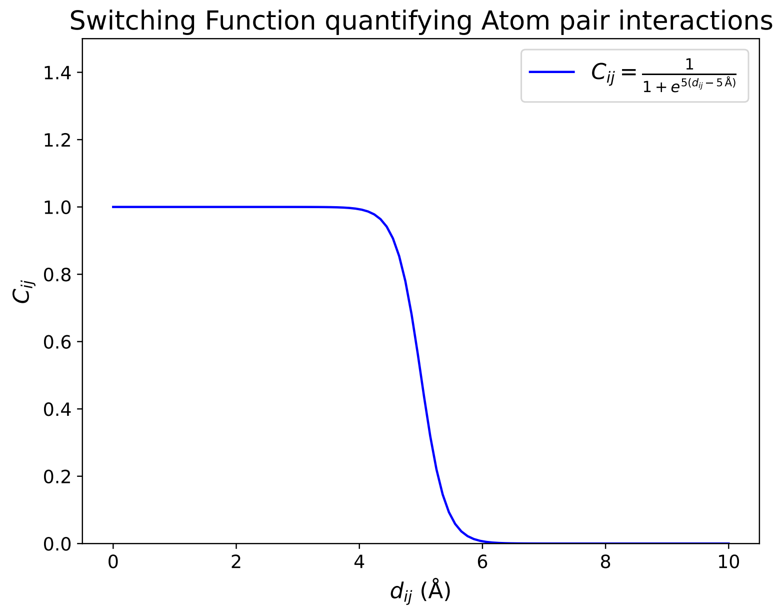

Figure S6: **Formula for how points are allocated when quantifying interactions through atom pairs**. The plot indicates that if the atom pair is within 4 Å, the interaction counts as 1 towards the contact sum, whilst between 4 Å to 6 Å, the interaction is partially contributes based on the shape of the curve.

$$
\begin{aligned}
&\textbf{Formula:}\\
&\sigma_{\Delta G} = RT \cdot \sqrt{\left(\frac{\sigma_s}{s}\right)^2 + \left(\frac{\sigma_c}{c}\right)^2}\\
&\textbf{Substitution:}\\
&\sigma_{\Delta G} = 0.5961 \cdot \sqrt{\left(\frac{1.31735}{21.77923}\right)^2 + \left(\frac{18.42319}{203.87089}\right)^2}\\
&= \boxed{0.06482 \text{ kcal/mol}}
\end{aligned}
\tag{S1}
$$

Eq. S1 shows the ΔG error calculation for acetyl group interactions at 15% WPG based on Eq. 11.

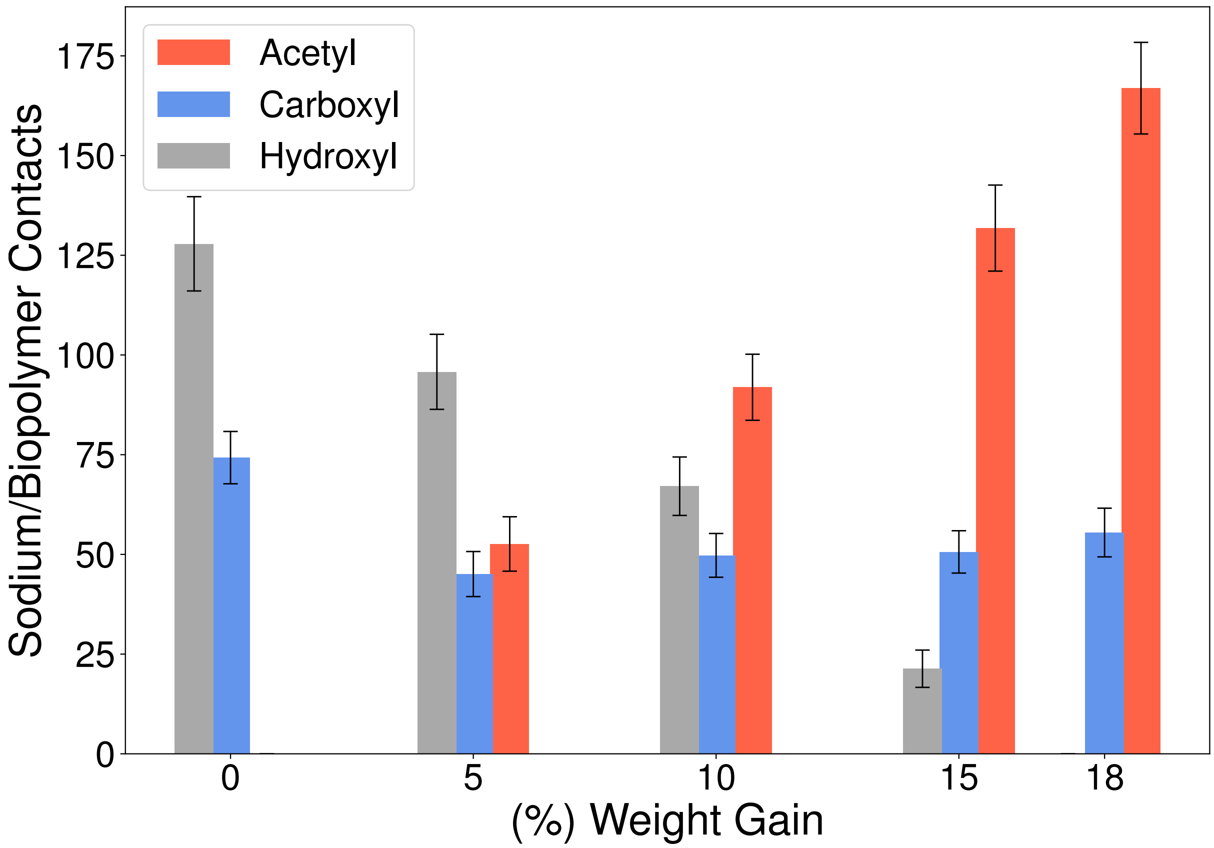

Figure S7: **Specific interactions between Na^+ and moieties on plant cell wall biopolymers.** The interactions for lignin and hemicellulose moieties (acetyl, carboxyl and hydroxyl) with Na^+ for each of the different acetylation degrees are quantified by Eq. 6 (Fig. S6). In this panel, we are measuring the contacts for the ”expanded” simulation set. Error estimates for each condition are a result of subdividing the 1 μs trajectory after 50 ns of equilibration into 19 50 ns chunks to determine the standard error.

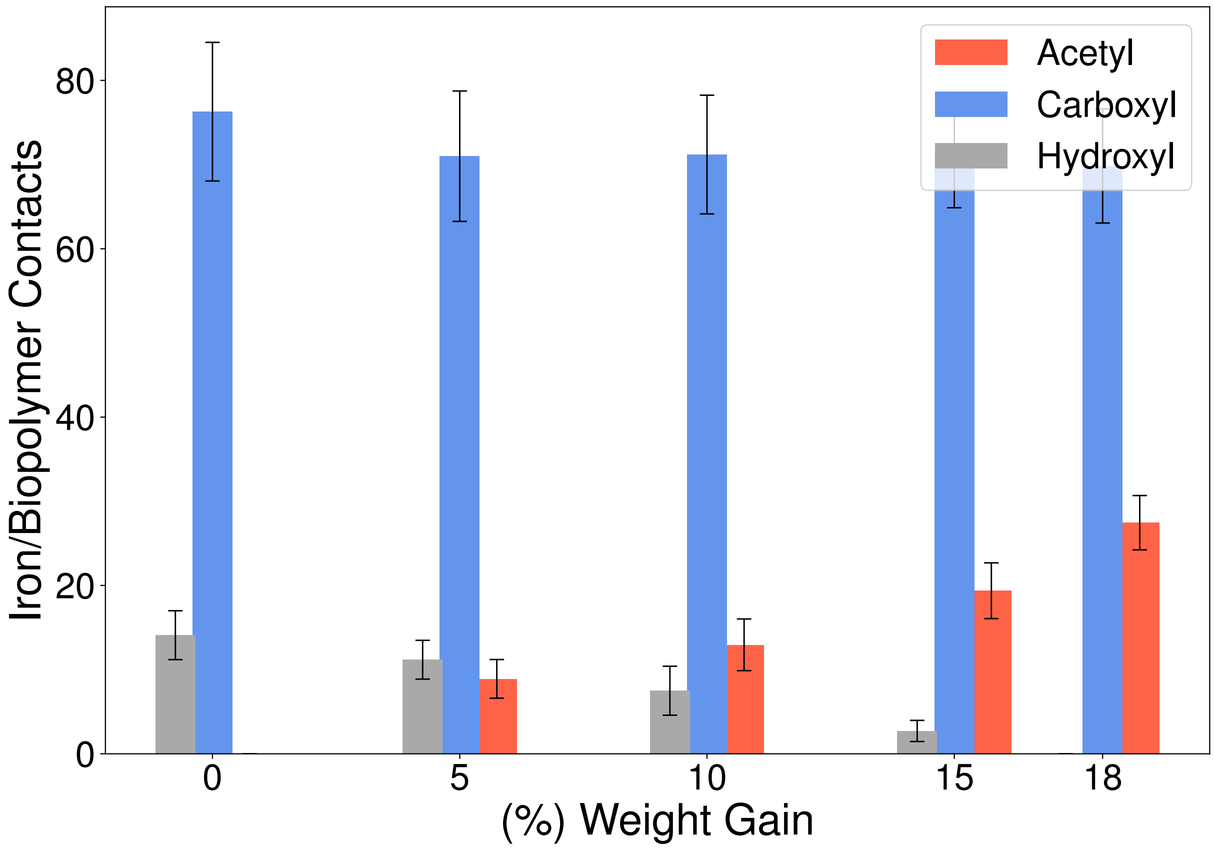

Figure S8: **Specific interactions between Fe^{3+} and moieties on plant cell wall biopolymers.** The interactions for lignin and hemicellulose moieties (acetyl, carboxyl and hydroxyl) with Na^+ for each of the different acetylation degrees are quantified by Eq. 6 (Fig. S6). In this panel, we are measuring the contacts for the "expanded" simulation set. Error estimates for each condition are a result of subdividing the 1 μs trajectory after 50 ns of equilibration into 19 50 ns chunks to determine the standard error.

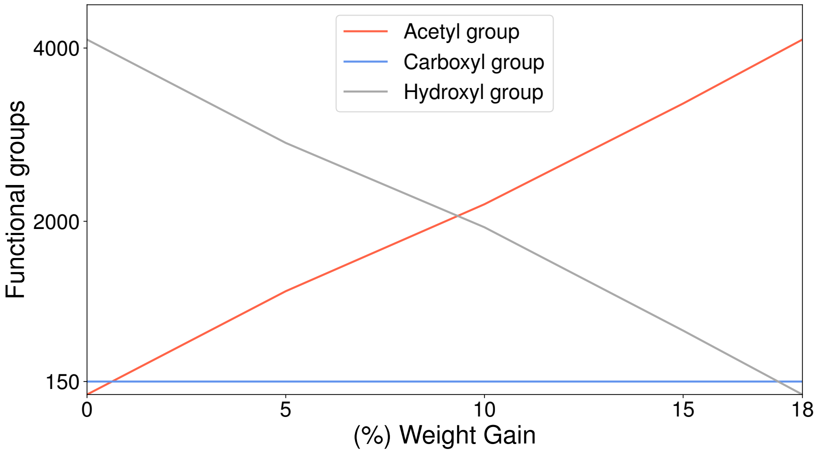

Figure S9: **Evolution of functional groups' quantities across the degrees of acetylation**. Acetyl groups replace hydroxyl groups as acetylation increases. The 150 carboxyl groups are retained across all molecular systems, and are exclusively on the hemicellulose.

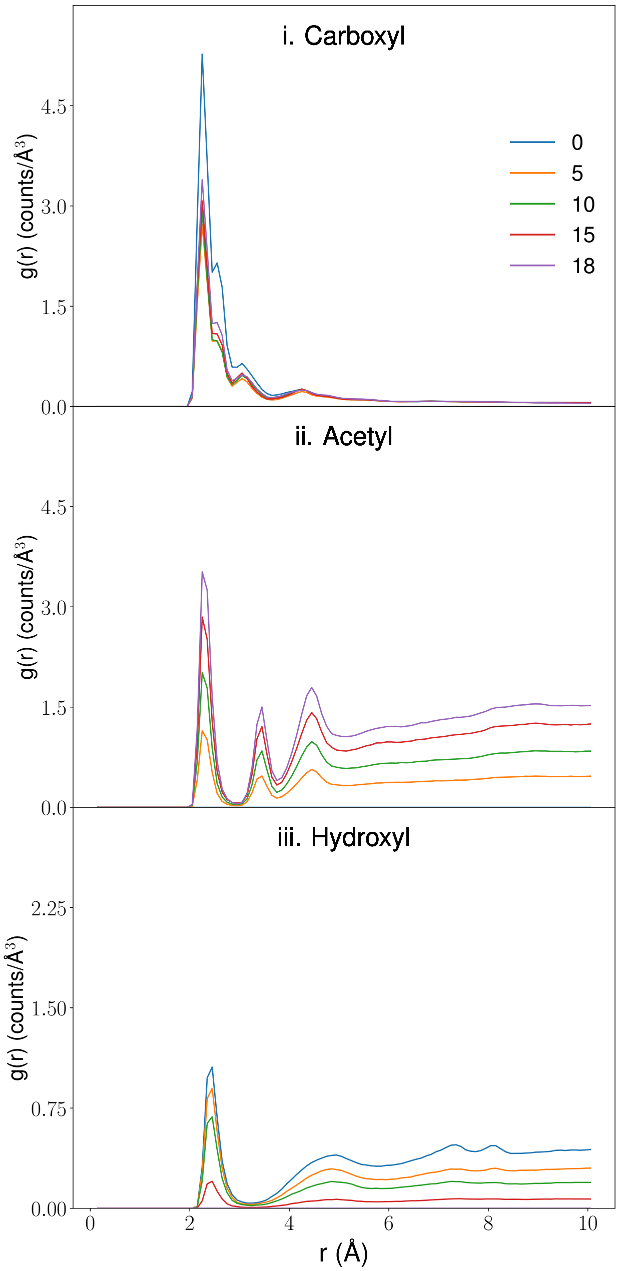

Figure S10: **Radial Distribution Function (RDF) profile for interactions between Na^+ ions and moieties on plant cell wall biopolymers in the 'expanded system'.** RDF plots show the density of i. Carboxyl group, ii. Acetyl group, and iii. Hydroxyl groups at distance r, within 10Å of the Na^+ ions. The densities have been tabulated at each acetylation degree.

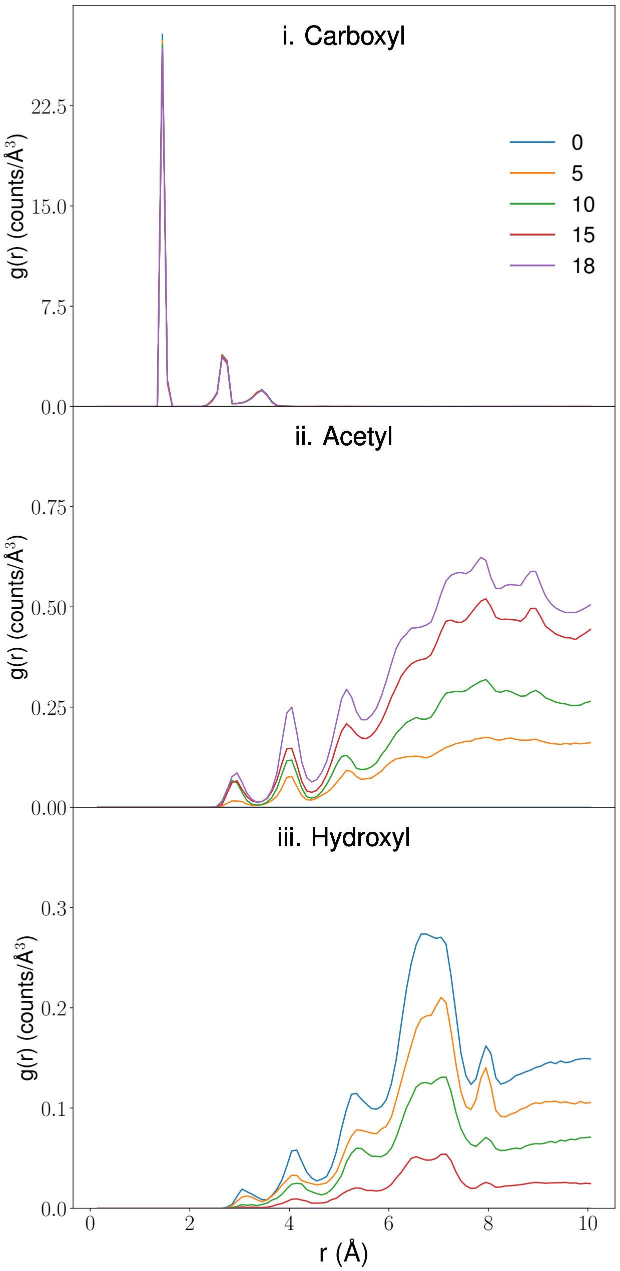

Figure S11: **Radial Distribution Function (RDF) profile for interactions between $\mathbf{Fe_3}^+$ ions and moieties on plant cell wall biopolymers in the 'expanded system'.** RDF plots show the density of i. Carboxyl group, ii. Acetyl group, and iii. Hydroxyl groups at distance r, within 10Å of the Fe^{3+} ions. The densities have been tabulated at each acetylation degree.

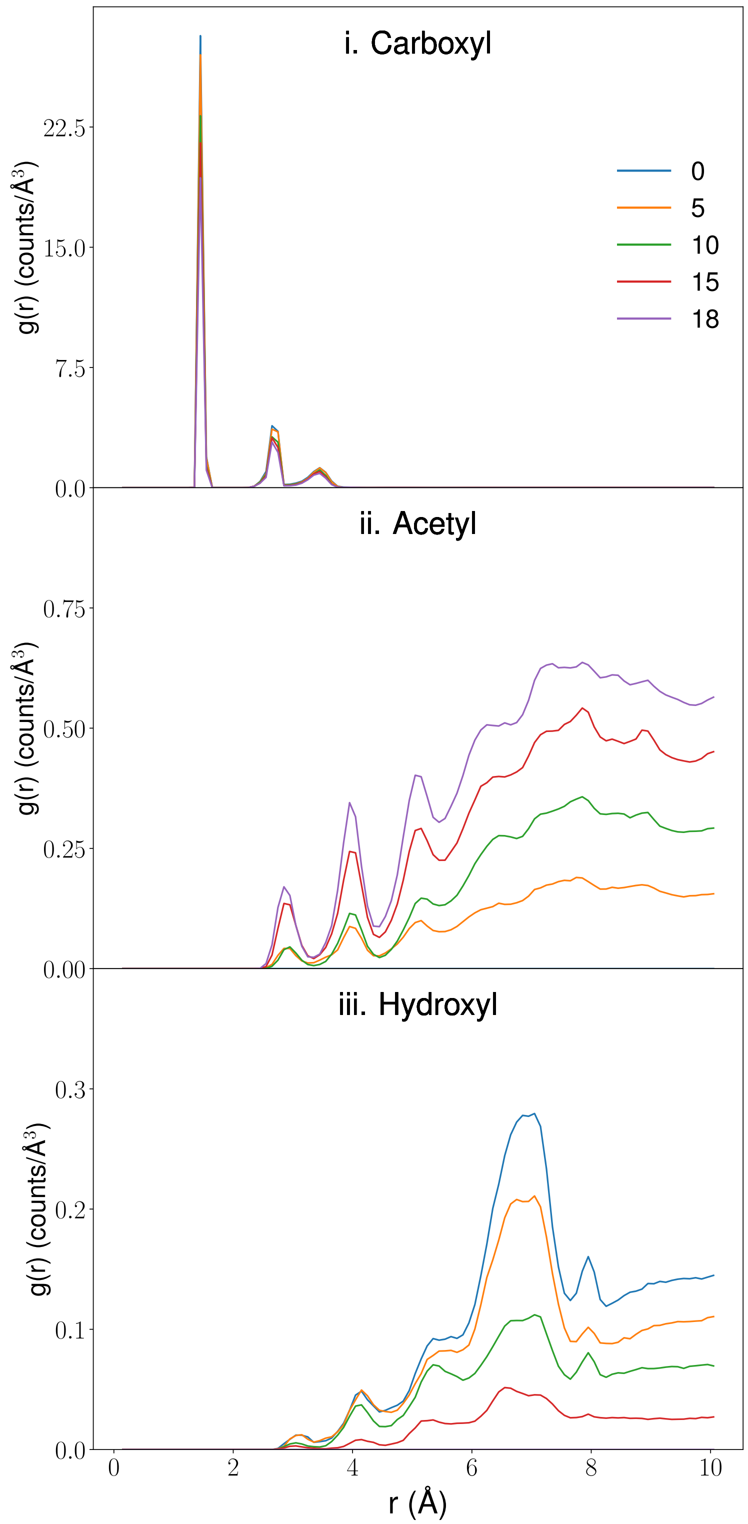

Figure S12: **Radial Distribution Function (RDF) profile for interactions between $Fe_3{}^+$ ions and moieties on plant cell wall biopolymers in the 'constant system'.** RDF plots show the density of i. Carboxyl group, ii. Acetyl group, and iii. Hydroxyl groups at distance r, within 10Å of the Fe^{3+} ions. The densities have been tabulated at each acetylation degree.

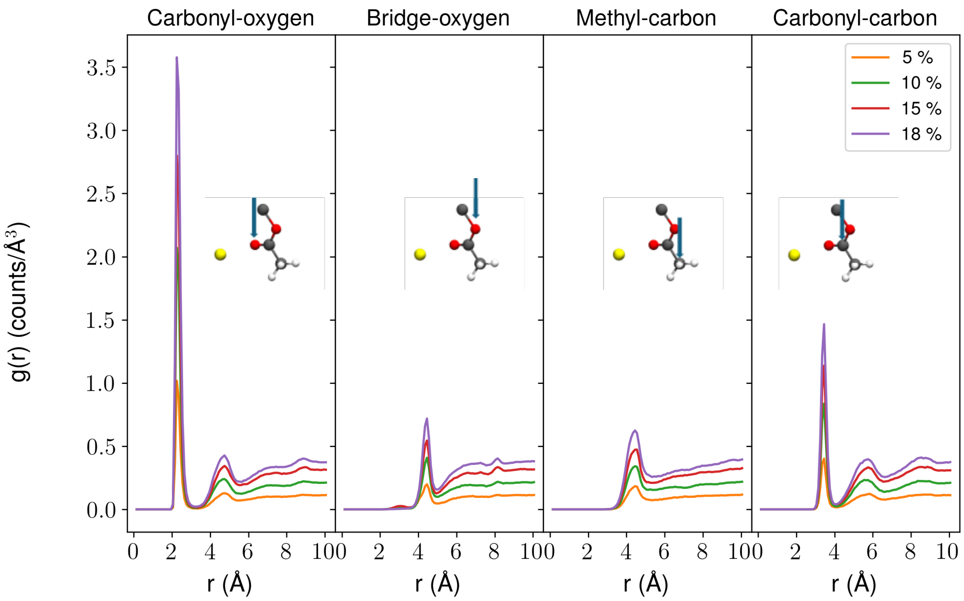

Figure S13: **Radial Distribution Function for individual atoms within acetyl group during interactions between Na^+ and acetyl groups** The figure explores which individual atom within the acetyl group is involved during the interaction with Na^+, providing greater insight into the chemical mechanism of this interaction.

Table S4: **Integrals for RDF values for interactions between Na^+ ions and acetyl, carboxyl and hydroxyl groups in the constant system, at varying degrees of acetylation**

Functional Group	0% WPG	5% WPG	10% WPG	15% WPG	18% WPG
Acetyl Group	0.00	18.6	20.9	21.8	21.3
Carboxyl Group	243	146	167	204	244
Hydroxyl Group	5.50	7.05	6.78	10.2	0.00

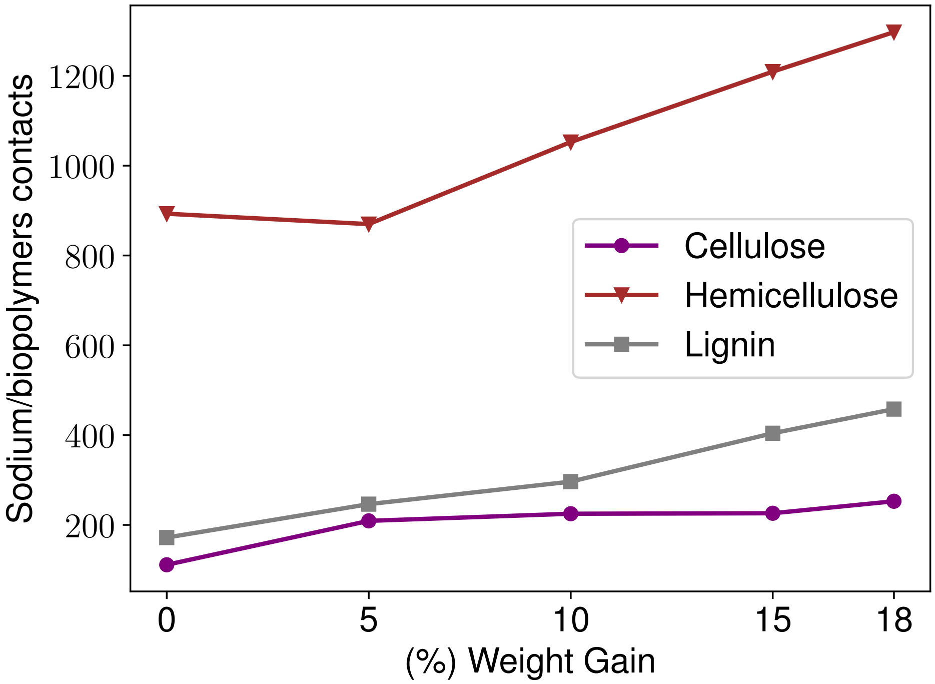

Figure S14: **Atom pair interactions between Na^+ ions and cell wall polymers (cellulose, hemicellulose, lignin)** The figure illustrates that hemicellulose polymers have the greatest number of interactions with Na^+ ions in the constant system, and acetylation causes this interaction to increase more significantly than Na^+ interactions with lignin and cellulose.

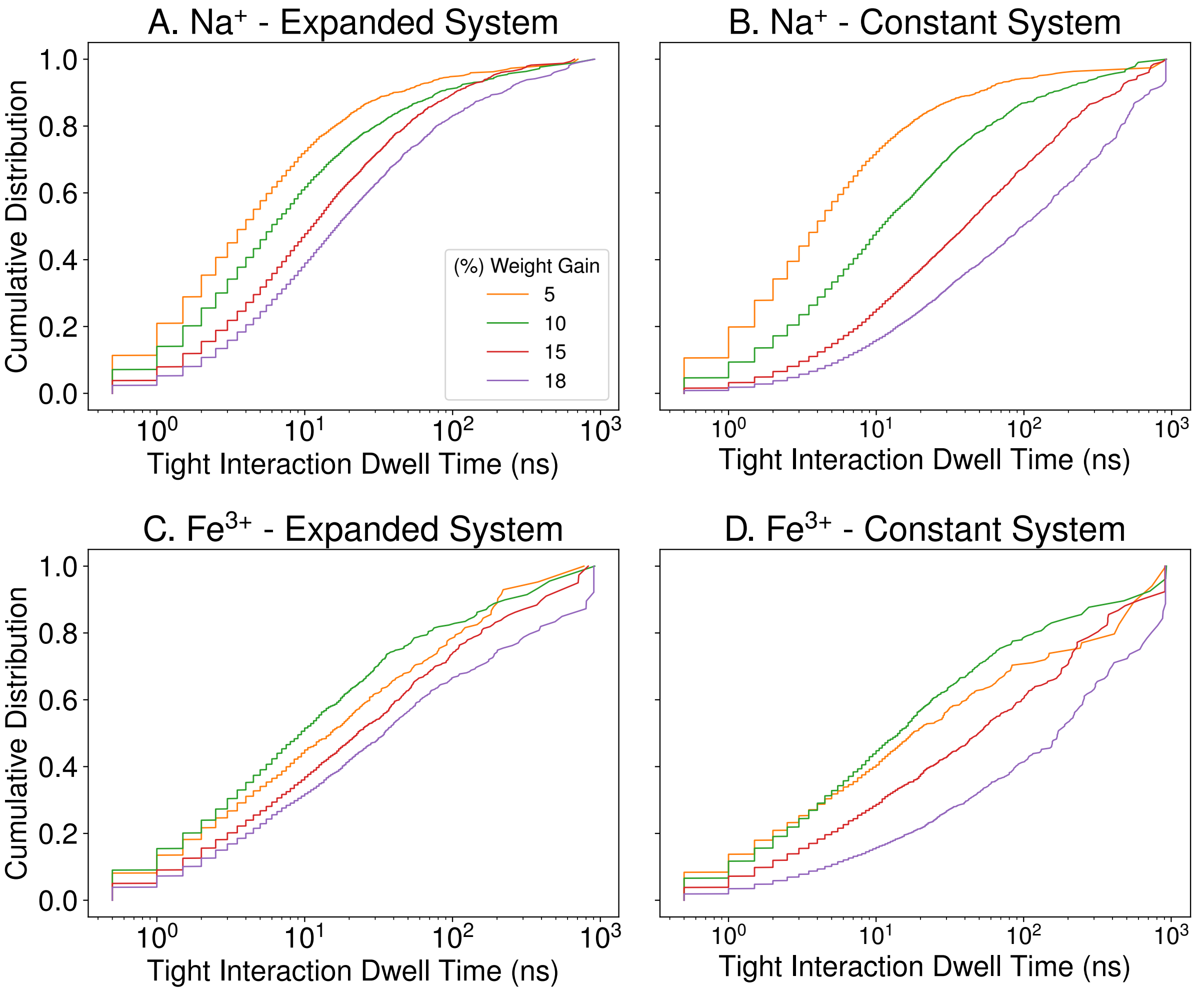

Figure S15: **Dwell time analysis of cation interactions with acetyl groups in 'expanded' and 'constant' systems at each acetylation degree.** The interaction dwell times between cations and acetyl groups are depicted, showing the dwell time duration against the proportion of interactions exhibiting this behavior. Quantifications were performed at each acetylation degree within the following systems: (A) expanded system neutralized by Na^+ ions, (B) constant system neutralized by Na^+ ions, (C) expanded system neutralized by Fe^{3+} ions, and (D) constant system neutralized by Fe^{3+} ions.

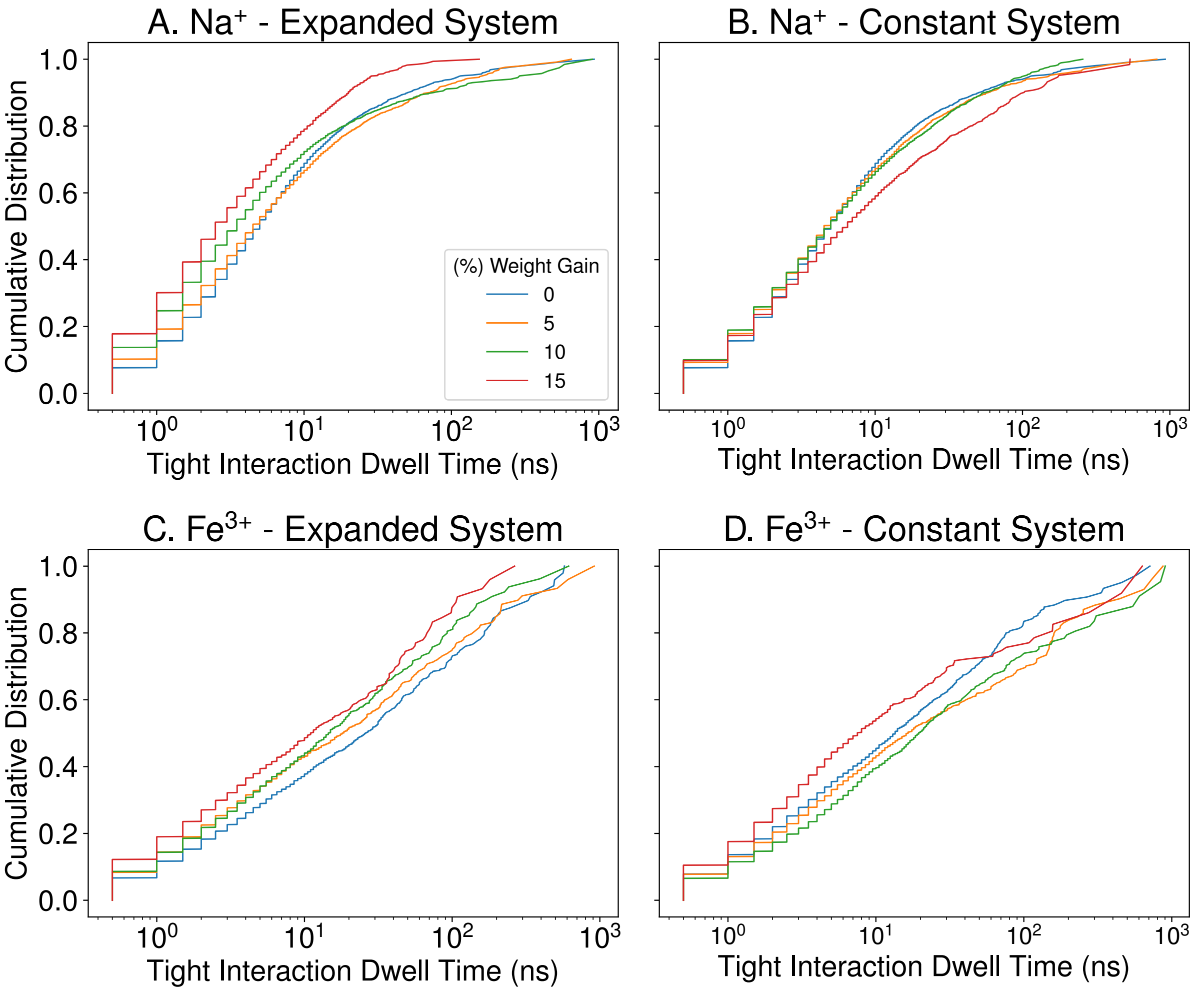

Figure S16: **Dwell time analysis of cation interactions with hydroxyl groups in 'expanded' and 'constant' systems at each acetylation degree.** The interaction dwell times between cations and hydroxyl groups are depicted, showing the dwell time duration against the proportion of interactions exhibiting this behavior. Quantifications were performed at each acetylation degree within the following systems: (A) expanded system neutralized by Na^+ ions, (B) constant system neutralized by Na^+ ions, (C) expanded system neutralized by Fe^{3+} ions, and (D) constant system neutralized by Fe^{3+} ions.

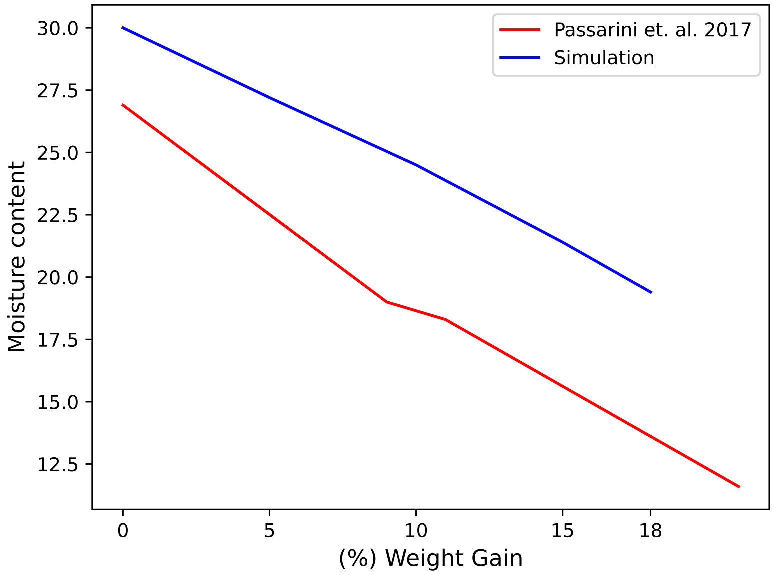

Figure S17: **Comparing equilibrium moisture content (EMC) change in the simulated model over the degrees of acetylation against previous literature data**. Passarini et al. (S1) used Differential scanning calorimetry to elucidate that acetylation reduces moisture content in the secondary plant cell wall. Our model follows a similar degree of reduction in moisture content over the degrees of acetylation, providing an additional layer of validation for our approach.

References

(S1) Passarini, L.; Zelinka, S. L.; Glass, S. V.; Hunt, C. G. Effect of Weight Percent Gain and Experimental Method on Fiber Saturation Point of Acetylated Wood Determined by Differential Scanning Calorimetry. *Wood Sci Technol* **2017**, *51*, 1291–1305, DOI: 10.1007/s00226-017-0963-0.
